# Blebbistatin reduces calcium buffering in cardiomyocytes: Consequences for cellular electrophysiology

**DOI:** 10.1113/JP287545

**Published:** 2025-08-25

**Authors:** Izzatullo Sobitov, Katharina Ritzenhoff, Marie Gaulrapp, Lea Becker, Aiste Liutkute, Fitzwilliam Seibertz, Funsho E. Fakuade, Fleur E. Mason, Niels Voigt

**Affiliations:** ^1^ Institute of Pharmacology and Toxicology, University Medical Center Göttingen Georg‐August University Göttingen Göttingen Germany; ^2^ DZHK (German Center for Cardiovascular Research) partner site Lower Saxony Germany; ^3^ Cluster of Excellence ‘Multiscale Bioimaging: from Molecular Machines to Networks of Excitable Cells’ (MBExC) University of Göttingen Göttingen Germany; ^4^ Department for Cardiothoracic and Vascular Surgery, University Medical Center Göttingen Georg‐August University Göttingen Göttingen Germany

**Keywords:** blebbistatin, calcium buffering, cardiac arrhythmia, excitation–contraction coupling

## Abstract

**Abstract:**

Blebbistatin is an excitation–contraction uncoupling agent commonly used in cardiac optical mapping; however, it has been reported to influence cardiac myofilament Ca^2+^ sensitivity. As primary contributors to Ca^2+^ buffering within cardiomyocytes, cardiac myofilaments play a critical role, and even minor disruptions in intracellular Ca^2+^ buffering significantly alter the free Ca^2+^ concentration. In this study, we investigated the effect of blebbistatin, a myosin II ATPase inhibitor, on intracellular Ca^2+^ buffering and cellular electrophysiology in induced pluripotent stem cell‐derived atrial cardiomyocytes. Simultaneous whole‐cell ruptured patch‐clamp and fluorescence microscopy techniques were used to assess intracellular Ca^2+^ handling, in addition to automated high‐throughput patch‐clamp to investigate ion channel function. Comprehensive analysis of Ca^2+^ buffering revealed that blebbistatin (10 µmol/l) causes a significant increase in buffer dissociation constant, suggesting decreased affinity of Ca^2+^ buffers. Furthermore, systolic and diastolic Ca^2+^ levels, sarcoplasmic reticulum Ca^2+^ leak and the incidence of spontaneous Ca^2+^ release events were significantly higher upon blebbistatin treatment. Although there was lack of impact on *I*_Na_ and *I*_Ca,L_ peak density, Ca^2+^‐dependent inactivation of *I*_Ca,L_ was significantly enhanced, and *I*_K1_ density was significantly smaller after blebbistatin. Importantly, these effects were reversed after chelation of intracellular Ca^2+^ with EGTA. Our observations indicate that blebbistatin reduces Ca^2+^ buffering, which, in turn, causes changes in cellular electrophysiology in cardiomyocytes.

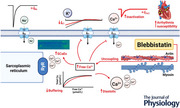

**Key points:**

- Intracellular Ca^2+^ buffering plays an important role in determining Ca^2+^ dynamics in cardiomyocytes.
- Blebbistatin, an excitation–contraction uncoupling agent widely used in experimental studies, decreases the affinity of intracellular Ca^2+^ buffers, as indicated by an increased buffer dissociation constant.
- Blebbistatin leads to higher systolic and diastolic Ca^2+^ levels and increased sarcoplasmic reticulum Ca^2+^ leak.
- Blebbistatin enhances L‐type Ca^2+^ current (*I*_Ca,L_) Ca^2+^‐dependent inactivation and reduces inward rectifier potassium current (*I*_K1_) density, while *I*_Na_ and *I*_Ca,L_ remain unchanged.
- The effects of blebbistatin are mitigated by chelation of intracellular Ca^2+^ with EGTA, showing that they are secondary to altered Ca^2+^ buffering.

## Graphical abstract

**Abstract figure legend** Blebbistatin reduces Ca^2+^ buffering in induced pluripotent stem cell‐derived atrial cardiomyocytes. Blebbistatin, a widely used excitation–contraction uncoupling agent, decreases calcium (Ca^2+^) buffer affinity (increased buffer dissociation constant), leading to elevated diastolic Ca^2+^ levels, increased sarcoplasmic reticulum Ca^2+^ leak and spontaneous Ca^2+^ release events. Additionally, blebbistatin exhibits no effect on peak sodium (*I*_Na_) and peak L‐type Ca^2+^ current (*I*_Ca,L_) densities. Nevertheless, blebbistatin enhances inactivation kinetics of *I*_Ca,L_ and reduces inward rectifier potassium current (*I*_K1_) density. Abbreviations: RyR, ryanodine receptor; SCaEs, spontaneous Ca^2+^ release events.

## Introduction

Blebbistatin, a myosin II ATPase inhibitor (Straight et al., 2003), has been used extensively in optical mapping research as an effective excitation–contraction (EC) uncoupling agent (Fedorov et al., 2007). However, recent evidence suggests that blebbistatin might increase arrhythmia susceptibility (Brack et al., 2013; Kappadan et al., 2020). Specifically, studies have shown that blebbistatin prolongs action potential duration and might indirectly modulate Ca^2+^ sensitivity, potentially impairing intracellular Ca^2+^ homeostasis (Baudenbacher et al., 2008; Fakuade et al., 2024).

Impaired Ca^2+^ buffering has emerged as a crucial factor contributing to the maintenance and progression of persistent atrial fibrillation (AF) (Fakuade et al., 2024). Importantly, as AF persists, it drives atrial remodelling (‘AF begets AF’) (Wijffels et al., 1995; Nattel et al., 2008) and significantly alters Ca^2+^ handling (Kneller, 2002; Vest et al., 2005; Voigt et al., 2014, 2012), making the disease self‐promoting and thus difficult to manage (Heijman et al., 2014; Joglar et al., 2024). Considering that ∼99% of intracellular Ca^2+^ is bound to buffers, even minor perturbations in the amount of Ca^2+^ buffered can have a major impact on free Ca^2+^ (Smith & Eisner, 2019), thereby influencing EC coupling. Both increased and reduced buffering capacity can increase arrhythmia susceptibility, as discussed recently in an editorial by Knollmann and Bers (2024).

In this study, we examine the direct effects of blebbistatin (10 µmol/l). We investigate whether blebbistatin selectively disrupts intracellular Ca^2+^ buffering, thereby inducing pro‐arrhythmic changes in Ca^2+^ handling and cellular electrophysiology.

## Methods

### Ethical approval

The study adheres to the principles of the *Declaration of Helsinki*, except for registration in a clinical trials database. Ethical approval for all protocols was granted by the Ethics Committee of the University Medical Center Göttingen (approval nos 10/9/15 and 15/2/20). Informed consent was obtained, and all research procedures were conducted in compliance with applicable guidelines and regulations.

### Atrial subtype‐directed differentiation of human induced pluripotent stem cells

The human induced pluripotent stem cell (hiPSC) line UMGi014‐C clone 14 (isWT1.14) was generated from dermal fibroblasts obtained from a healthy 31‐year‐old male donor. The cells were cultured in feeder‐free conditions and reprogrammed using the integration‐free CytoTune iPS 2.0 Sendai Reprogramming Kit (Thermo Fisher Scientific) using the reprogramming factors OCT4, KLF4, SOX2 and c‐MYC. As reported in previous studies on this cell line, assessments of pluripotency and karyotype confirmed the absence of abnormalities or chromosomal instability (Rössler et al., 2021). Subtype‐directed differentiation of iPSC‐atrial cardiomyocytes (iPSC‐aCM) was achieved by following a previously published protocol (Cyganek et al., 2018; Kleinsorge & Cyganek, 2020).

Cells were kept at 37°C with 5% CO_2_. For the purpose of stem cell maintenance and further differentiation, iPSCs were cultured with StemMACS iPS‐Brew XF (Miltenyi Biotec) until confluency of the monolayer was reached. Subsequently, mesodermal induction (designated as day 0) was started in ‘differentiation medium’ containing 4 µmol/l CHIR99021 in RPMI 1640 Medium, GlutaMAX™ Supplement (Thermo Fisher Scientific), 0.5 mg/ml human recombinant albumin and 0.2 mg/ml l‐ascorbic acid 2‐phosphate (all from Sigma‐Aldrich).

After 48 h, cardiac differentiation was initiated by application of 5 µmol/l IWP2 (Wnt Antagonist II, Merck) in ‘differentiation medium’ for another 48 h. To promote atrial subtype specification, 1 µmol/l retinoic acid (Sigma‐Aldrich) was added on days 3 and 5. Spontaneous cellular contractions typically emerged between days 10 and 14.

Metabolic lactate selection was initiated to enhance cardiac population purity. For this purpose, cells were cultured for ≤7 days in a ‘selection medium’ comprising RPMI 1640 without glucose (Thermo Fisher Scientific), 0.5 mg/ml human recombinant albumin, 0.2 mg/ml l‐ascorbic acid 2‐phosphate and 4 mM lactate (all from Sigma‐Aldrich). Later, cells were maintained in a ‘culture medium’ containing RPMI 1640 Medium, GlutaMAX™ supplemented with 2% B27 (both from Thermo Fisher Scientific). Following lactate selection, cells were maintained in ‘culture medium’ until further experiments or manipulations were performed.

### Coverslip preparation

Flasks with iPSC‐aCM (days 35–40) were washed with Dulbecco's phosphate‐buffered saline Ca/Mg^−/−^ (Gibco/Thermo Fisher Scientific) and incubated with Trypsin‐EDTA (0.25%) Phenol Red+ (Gibco/Thermo Fisher Scientific) for ≤15 min. Digestion was blocked with fetal bovine serum (Gibco/Thermo Fisher Scientific) with a ratio of 1:1. Next, cells were resuspended following centrifugation at 4°C at 100*g* for 10 min and counted using a counting chamber (Neubauer improved, Paul Marienfeld GmbH & Co. KG). Then, cells were plated onto glass coverslips (10mm diameter Menzel coverslip, Thermo Fisher) precoated with 1:60 Matrigel^®^ (Corning). The plating density for experiments was 15 000 cells/cm^2^. Cells were maintained in the ‘culture medium’ at 37°C with 5% CO_2_.

### Simultaneous patch‐clamp and intracellular Ca^2+^ measurements

iPSC‐aCM plated on Matrigel‐coated coverslips were transferred to a 37 ± 0.5°C preheated chamber and were continuously superfused with bath solution (in mmol/l: 4‐aminopyridine, 5; BaCl_2_, 0.1; CaCl_2_, 2; glucose, 10; Hepes, 10; KCl, 4; MgCl_2_, 1; NaCl, 140; and probenecid, 2; pH adjusted to 7.35 with NaOH). The pipette solution (in mmol/l: EGTA, 0.02; GTP‐Tris, 0.1; Hepes, 10; potassium aspartate, 92; KCl, 48; MgATP, 1; Na_2_ATP, 4; pH adjusted to 7.2 with KOH) contained fluo‐3 pentapotassium salt, 0.1 mmol/l (Thermo Scientific). Intracellular Ca^2+^ was quantified using fluo‐3 acetoxymethyl ester (Fluo‐3 AM; 10 µmol/l; 10 min incubation followed by 30 min de‐esterification).

Simultaneous measurements of membrane currents and intracellular Ca^2+^ ([Ca^2+^]_i_) were performed using a voltage‐clamp protocol in the whole‐cell ruptured patch configuration. Membrane currents were recorded at 0.5 Hz stimulation with a 600 ms ramp pulse (to inactivate the fast Na^+^ current) from −80 to −40 mV, followed by a 100 ms voltage step to +10 mV, which activated Ca^2+^ current, triggering cytosolic Ca^2+^ transients. Data acquisition and analysis were performed using pClamp Software (v.10.7, Molecular Devices). Membrane currents were normalised to membrane capacitance values and expressed as current density (in picoamperes per picofarad, pA/pF). Borosilicate glass microelectrodes with tip resistance of 3–7 MΩ were used. Recordings with a seal resistance of <1 GΩ were excluded. Cells were incubated for 20 min with 10 µmol/l blebbistatin with a final DMSO concentration of 0.01 %. Control solutions were prepared by adding an equivalent amount of DMSO and used consistently in all experiments.

The [Ca^2+^]_i_ was quantified by exciting fluo‐3 at 488 nm and detecting emitted light (>520 nm), with subsequent estimation of [Ca^2+^]_i_ using the formula:

$${\left[Ca^{2+}\right]}_{i}=K_{d}\left(\frac{F}{F_{\max}-F}\right)$$

where *K*_d_ is the dissociation constant of fluo‐3 (864 nM), *F* is the fluo‐3 fluorescence, and *F*_max_ is the Ca^2+^‐saturated fluorescence obtained at the end of each experiment. Ca^2+^ transients were analysed by averaging 10 consecutive traces (Voigt et al., 2014, 2012).

Sarcoplasmic reticulum (SR) Ca^2+^ content and cytosolic Ca^2+^ buffering were quantified by applying a high concentration of caffeine (10 mmol/l), as previously described (Díaz et al., 1997, 2001). In brief, the integral of the caffeine‐induced Na^+^–Ca^2+^ exchanger (NCX) current was plotted against free [Ca^2+^]_i_, as determined from the decay phase of the caffeine‐induced Ca^2+^ transient. Then, data were fitted with a Michaelis–Menten buffer curve (Díaz et al., 1997, 2001; Fakuade et al., 2024; Smith & Eisner, 2019; Trafford et al., 1999), as follows:

$${\left[Ca^{2+}\right]}_{\mathrm{total}}=a+\frac{B_{\max}{\left[Ca^{2+}\right]}_{i}}{K_{d}+{\left[Ca^{2+}\right]}_{i}}$$

where *a* is the amount of Ca^2+^ bound to the buffer in diastole, *K*_d_ is the dissociation constant, and *B*_max_ is the maximum buffering capacity.

Ca^2+^ sparks in iPSC‐aCM were measured after incubation with fluo‐4 AM (final concentration of 5 µmol/l) for 15 min, followed by a 15 min de‐esterification period in dye‐free Tyrode solution. Imaging was performed on a confocal microscope system (LSM‐5, Zeiss). Confocal line scans were performed using a ×40 oil objective (512 pixels, 37.5 µm, 10 000 cycles, pinhole 67 µm) with prior field stimulation at 2.5 Hz for 30 s. Images were acquired using Zen 2009 acquisition software and analysed using the ImageJ SparkMaster plug‐in (Picht et al., 2007). The SR Ca^2+^ leak was calculated from Ca^2+^ spark frequency and Ca^2+^ spark size, as previously described (Fischer et al., 2015).

### Automated high‐throughput patch clamp

First, iPSC‐aCM (days 40–45) were incubated for 20 min with 10 µmol/l blebbistatin or DMSO. In the following dissociation steps, all solutions contained 10 µmol/l blebbistatin or DMSO, accordingly. This was done in order to maintain the effect of blebbistatin. To begin the cellular dissociation process, cells were washed with Dulbecco's phosphate‐buffered saline Ca/Mg^−/−^ (Gibco/Thermo Fisher Scientific) and briefly incubated with Versene™ (Gibco/Thermo Fisher Scientific) for ≤1 min. Then, cells were digested using TrypLE Express Enzyme (1×) without Phenol Red (Gibco/Thermo Fisher Scientific) for 30–45 min, until they were detached and dissociated into small clumps. Digestion was blocked by fetal bovine serum (Gibco/Thermo Fisher Scientific) with a ratio of 1:1. After centrifugation at 4°C at 100 *g* for 7 min, cells were resuspended in Hanks’ balanced salt solution Ca/Mg^−/−^ (Gibco/Thermo Fisher Scientific) and kept at 4°C for automated patch‐clamp experiments.

Measurement of currents was performed using an automated patch‐clamp machine, SyncroPatch 384 (Nanion Technologies). A negative pressure of −300 mbar was used to catch the cells, and additional pulses of negative pressure of −250 and −300 mbar, respectively, were used to gain whole‐cell access. PatchControl 384 (Nanion Technologies) software was used for data acquisition, and Biomek Software (Beckman Coulter) was used for experimental design. Measurements were performed on thin borosilicate glass 384‐well planar chips (1× S‐type NPC‐384T, Nanion Technologies) (Seibertz et al., 2022; Seibertz, Rubio et al., 2023). Recordings with a seal resistance of <500 MΩ were excluded.

Experiments were started by filling up the chip wells with 30 µl of divalent‐free solution (DVF; in mmol/l: NaCl, 140; KCl, 4; Hepes, 10; and glucose 5; pH adjusted to 7.4 with NaOH). Then, the pipette solution for *I*_Na_ and *I*_Ca,L_ (CsF internal; in mmol/l: CsF, 110; NaCl, 10; CsCl, 10; and Hepes, 10; pH adjusted to 7.2 with CsOH) was used to generate electrical contact. Subsequently, 40 µl of seal enhancer solution (SE NMDG; in mmol/l: NaCl, 80; NMDG, 60; KCl, 4; MgCl_2_, 1; CaCl_2_, 10; Hepes, 10; and glucose, 5; pH adjusted to 7.4 with HCl) was added. After this, the previous solution was exchanged to final bath solution (NMDG 140; mmol/l: NMDG, 140; KCl, 4; MgCl_2_, 1; CaCl_2_, 2; Hepes, 10; and glucose, 5; pH adjusted to 7.4 with HCl).

After acquisition of *I*_Na_ and *I*_Ca,L_ recordings, the pipette solution was exchanged to facilitate the recording of *I*_K1_ using a KF‐based pipette solution (KF internal; in mmol/l: KF, 110; NaCl, 10; KCl, 10; and Hepes 10; pH adjusted to 7.2 with KOH). The *I*_K1_ currents were measured with a 1000 ms ramp pulse from −100 to +40 mV. The current was recorded continuously during the sequential exchange of the bath solutions. Initially, the bath solution was NMDG 60 (in mmol/l: NaCl, 80; NMDG, 60; KCl, 4; MgCl_2_, 1; CaCl_2_, 2; Hepes, 10; and glucose, 5; pH adjusted to 7.4 with HCl). Then, the bath solution was replaced with high‐K^+^‐containing NMDG 60 (20 mmol/l KCl), and finally, with Ba^2+^‐containing NMDG 60 solution (1 mmol/l BaCl_2_) to achieve subsequent *I*_K1_ block. Inward rectifier currents that were sensitive to Ba^2+^ block following the application of a high‐K^+^‐containing bath solution were defined as *I*_K1_ and included in the dataset (Dobrev et al., 2005; Voigt et al., 2010, 2013).

Where indicated, EGTA (10 mmol/l) was added to the pipette solution (both CsF and KF internal solutions) as an intracellular Ca^2+^ chelator. All measurements were conducted at room temperature, allowing Ca^2+^‐ and fluoride‐assisted seal formation (Milligan et al., 2009; Seibertz et al., 2022). DataControl 384 (Nanion Technologies) software was used for data analysis.

### Statistical analysis

Data distribution was initially assessed using the Shapiro–Wilk test. Normally distributed data were compared using Student's unpaired two‐tailed *t* test, whereas non‐normally distributed data were analysed using the Mann–Whitney *U* test. Data are presented as the mean ± SD. A *P*‐value of <0.05 was considered statistically significant.

## Results

### Blebbistatin reduces Ca^2+^ buffering in iPSC‐aCM

**Figure 1:**
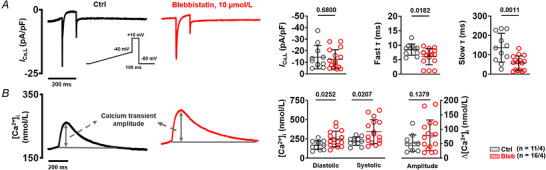
*I*_Ca,L_ and Ca^2+^ transients in induced pluripotent stem cell‐derived atrial cardiomyocytes *A*, representative traces of *I*_Ca,L_ with voltage‐clamp protocol (inset) and quantification (mean ± SD) of peak *I*_Ca,L_ amplitude and biphasic (fast and slow) inactivation kinetics. *B*, representative traces of CaT (fluo‐3 AM) and quantification (mean ± SD) of diastolic and systolic [Ca^2+^]_i_ and CaT amplitude. Cells were pretreated with blebbistatin (10 µmol/l). Comparisons made using Student's unpaired *t* test or the Mann–Whitney *U* test. *P*‐values are *versus* Ctrl. *n*/*N* = induced pluripotent stem cell‐derived atrial cardiomyocytes/batches. Abbreviations: Bleb, blebbistatin; CaT, Ca^2+^ transient; Ctrl, control.

Intracellular Ca^2+^ handling was investigated using simultaneous whole‐cell ruptured patch‐clamp electrophysiology and epifluorescence measurements. Interestingly, no significant difference in *I*_Ca,L_ peak density was observed between the blebbistatin‐treated group and the control (Ctrl) group (Fig. 1*A*). However, both diastolic Ca^2+^ levels (Ctrl, 168.3 ± 57.06 nmol/l, *n*/*N* myocytes/batches = 11/4 *versus* blebbistatin, 248.6 ± 100.9 nmol/l, *n*/*N* = 16/4; *P* = 0.0252) and systolic Ca^2+^ levels (Ctrl, 220.2 ± 54.42 nmol/l *versus* blebbistatin, 342.0 ± 156.2 nmol/l; *P* = 0.0207) were significantly higher in treated cells (Fig. 1*B*).

**Figure 2:**
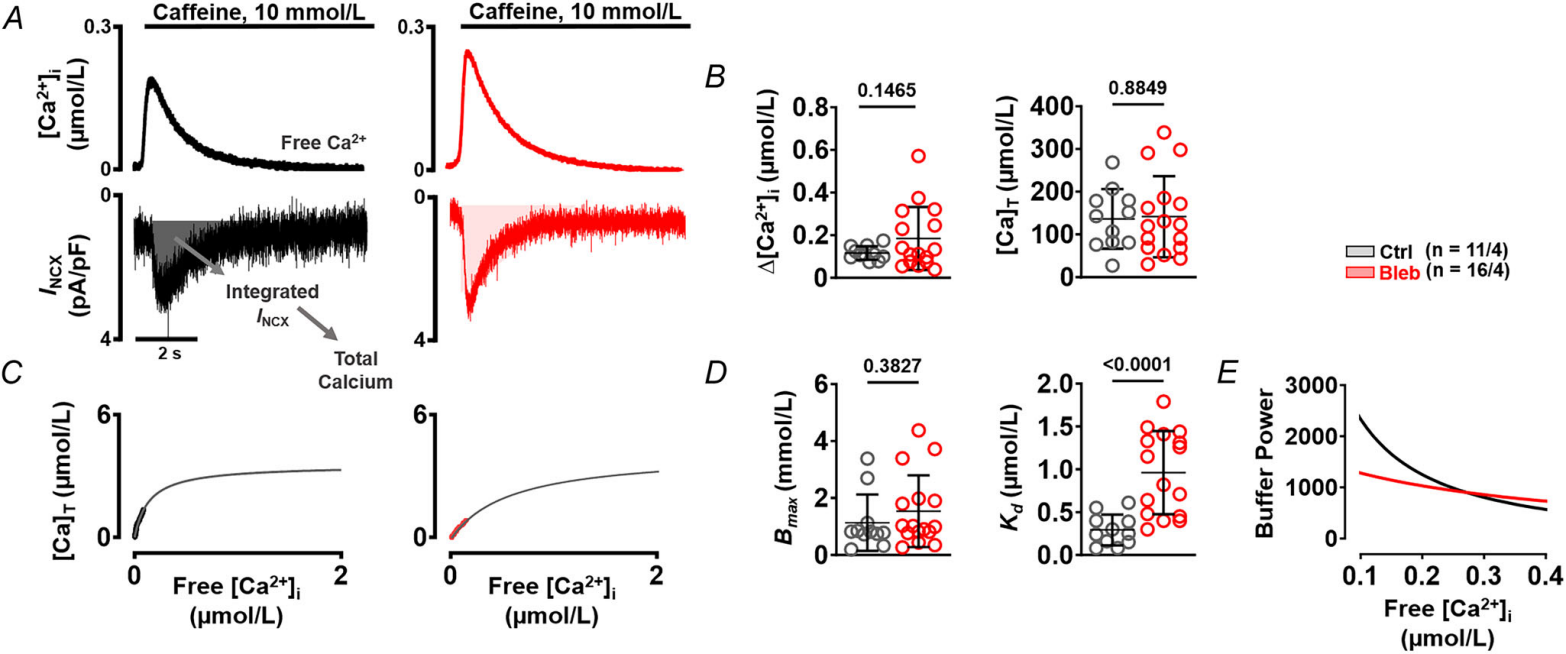
Intracellular Ca^2+^ buffering in induced pluripotent stem cell‐derived atrial cardiomyocytes *A*, representative traces of caffeine‐induced Ca^2+^ transients (cCaT) (free [Ca^2+^]_i_, top traces) and corresponding sodium–calcium exchanger currents (*I*_NCX_, bottom traces) integrated cumulatively to provide an index of total Ca^2+^. *B*, quantification (mean ± SD) of cCaT amplitude (left) and total calcium, [Ca]_T_ (right). *C*, representative buffer curves demonstrating the relationship between free [Ca^2+^]_i_ and total calcium, [Ca]_T_, fitted with a hyperbolic function. *D*, maximum buffer capacity (*B*_max_, left) and buffer dissociation constant (*K*_d_, right) (both mean ± SD). *E*, mean buffer power as a function of free [Ca^2+^]_i_, calculated from individual total buffer curves. Cells were pretreated with blebbistatin (Bleb, 10 µmol/l). Comparisons were made using Student's unpaired *t* test or the Mann–Whitney *U* test. *P*‐values are *versus* Ctrl. *n*/*N* = induced pluripotent stem cell‐derived atrial cardiomyocytes/batches. Abbreviations: Bleb, blebbistatin; *B_max_*, maximum buffering capacity; *K_d_*, dissociation constant; Ctrl, control.

After 3 min of stimulation, iPSC‐aCM were held at −80 mV, and 10 mmol/l caffeine was applied to induce complete Ca^2+^ release from the SR. The amplitude of the resulting caffeine‐induced Ca^2+^ transient (cCaT), representing the ‘free’ cytosolic Ca^2^⁺, was not significantly different in the blebbistatin‐treated group *versus* control, nor was the ‘total’ Ca^2+^, determined by integrating the inward NCX current and normalising to cell volume (Fig. 2*A* and *B*).

In order to estimate Ca^2+^ buffering properties, the total calcium concentration was plotted against cytosolic free Ca^2+^ during the decay of the cCaT (Diaz et al., 2001; Trafford et al., 1999). The resulting data were fitted with a Michaelis–Menten buffer equation (Fig. 2*C*) to estimate the maximum buffering capacity (*B*_max_) and buffer dissociation constant (*K*_d_) (Smith & Eisner, 2019). Although *B*_max_ was comparable between the groups, *K*_d_ was significantly higher in blebbistatin‐treated cells (Fig. 2*D*), indicating decreased affinity of intracellular Ca^2+^ buffers. Corrections were made for the contribution of fluo‐3, to ensure accurate estimates of Ca^2+^ buffering properties (Diaz et al., 2001; Trafford et al., 1999).

### Blebbistatin does not affect cytosolic Ca^2+^ removal

**Figure 3:**
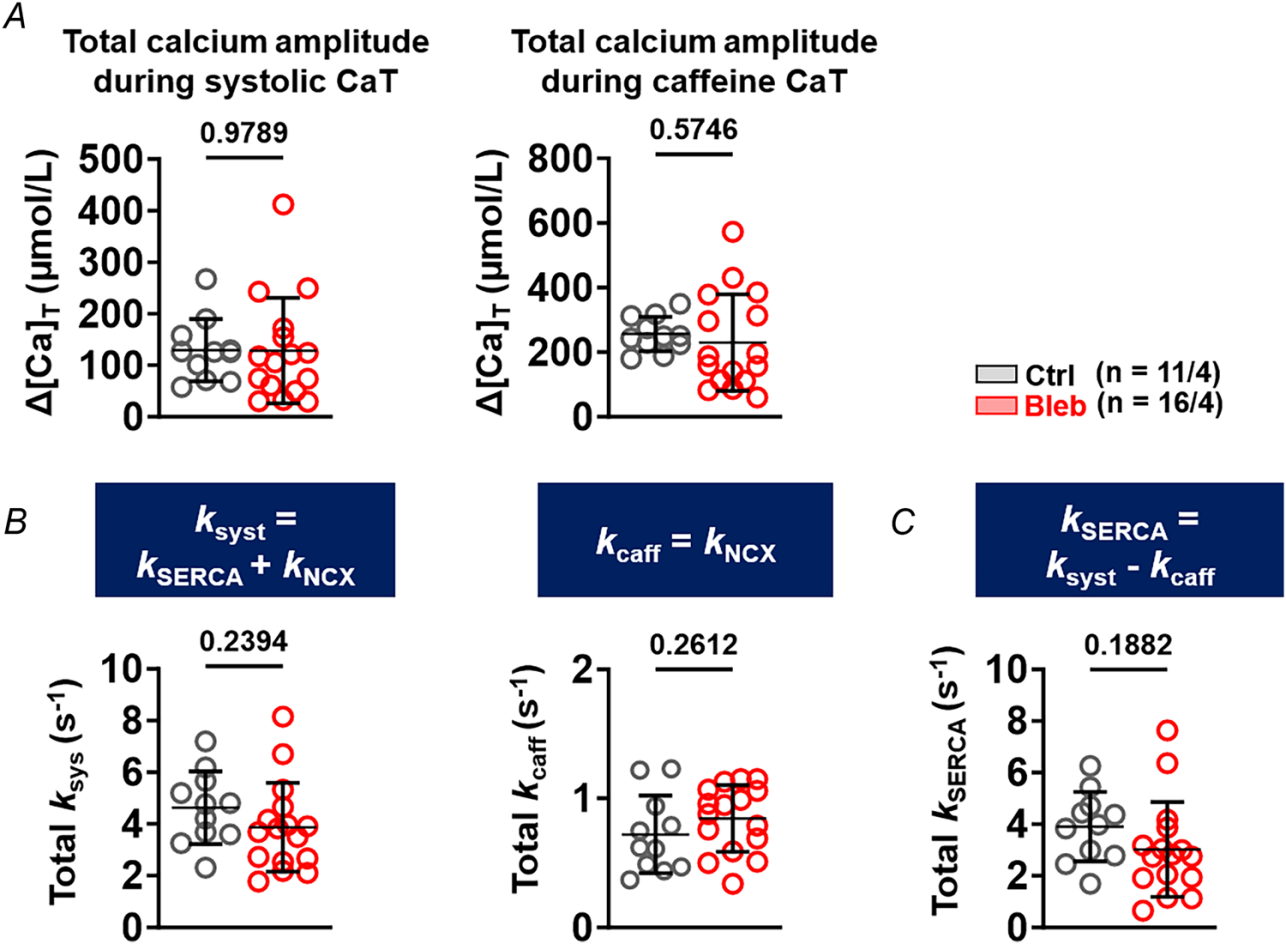
Decay of total Ca^2+^ in induced pluripotent stem cell‐derived atrial cardiomyocytes *A*, quantification (mean ± SD) of total Ca^2+^ ([Ca]_T_) amplitude during systolic CaT (left) and caffeine‐induced Ca^2+^ transient (cCaT, right). *B*, decay constant of total systolic CaT (Total *k*_sys_, left) and cCaT (total *k*_caff_, right) (both mean ± SD). *C*, total *k*_SERCA_ (mean ± SD; calculated by subtracting total *k*_caff_ from total *k*_sys_ in *B*). Cells were pretreated with blebbistatin (Bleb, 10 µmol/l). Comparisons made using Student's unpaired *t* test or the Mann–Whitney *U* test. *P*‐values versus Ctrl indicated. *n*/*N* = induced pluripotent stem cell‐derived atrial cardiomyocytes/batches. Abbreviations: Caff, caffeine; CaT, Ca^2+^ transient; SERCA, sarco/endoplasmic reticulum Ca^2+^ ATPase; Sys, systolic

To investigate cytosolic Ca^2+^ removal, the total Ca^2+^ concentration during the decay phase of the systolic and caffeine‐induced CaTs was calculated using the *B*_max_ and *K*_d_, and exponential curves were fitted to estimate the rate constant of decay (total *k*_sys_ and total *k*_caff_, respectively) (Diaz et al., 2001; Fakuade et al., 2024; Jung et al., 2022; Trafford et al., 1999). Both total *k*_sys_ and total *k*_caff_ were comparable in blebbistatin‐treated *versus* control groups (Fig. 3*B*). To determine the activity of the sarco‐endoplasmic reticulum Ca^2+^ ATPase (SERCA), total *k*_caff_ was subtracted from total *k*_sys_ and was found to be comparable in both groups (total *k*_SERCA_) (Fig. 3*B*). Thus, the findings suggest that blebbistatin alters Ca^2+^ buffering while leaving cytosolic Ca^2+^‐removal processes unaltered.

### Reduced Ca^2+^ buffering by blebbistatin increases the frequency of Ca^2+^ sparks

**Figure 4:**
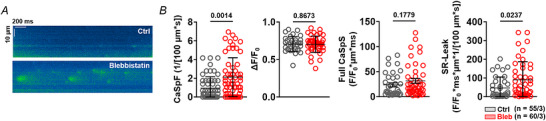
Incidence of Ca^2+^ sparks in induced pluripotent stem cell‐derived atrial cardiomyocytes *A*, representative confocal line scans of iPSC‐aCM, showing spontaneous SR Ca^2+^ release events as Ca^2+^ sparks in the presence and absence of blebbistatin (Bleb, 10 µmol/l) in iPSC‐aCM. *B*, quantification (mean ± SD) of Ca^2+^ spark frequency, Ca^2+^ spark amplitude, full Ca^2+^ spark size and SR Ca^2+^ leak. Comparisons were made using Student's unpaired *t* test or the Mann–Whitney *U* test. *P*‐values *versus* Ctrl are indicated. *n*/*N* = induced pluripotent stem cell‐derived atrial cardiomyocytes/batches. Abbreviations: Bleb, blebbistatin; CaSpF, Ca^2+^ spark frequency; CaSpS ‐ Ca^2+^ spark size; Ctrl, control; SR, sarcoplasmic reticulum.

Increased diastolic Ca^2+^ levels and altered Ca^2+^ buffering are known contributors to cardiac arrhythmias (Baudenbacher et al., 2008; Jung et al., 2022; Schober et al., 2012). We recently showed that application of blebbistatin, to modulate Ca^2+^ sensitivity indirectly in Langendorff‐perfused mouse hearts, causes an increase in arrhythmia inducibility (Fakuade et al., 2024). To investigate whether this effect extends to the occurrence of diastolic spontaneous Ca^2+^ release events (SCaEs), Ca^2+^ sparks in iPSC‐aCM were measured and analysed after blebbistatin treatment. Confocal line scans revealed both increased Ca^2+^ spark frequency (CaSpF) and significantly higher SR Ca^2+^ leak in blebbistatin‐treated cells, in comparison to control cells (Fig. 4).

### Reduced Ca^2+^ buffering by blebbistatin alters cellular electrophysiology

**Figure 5:**
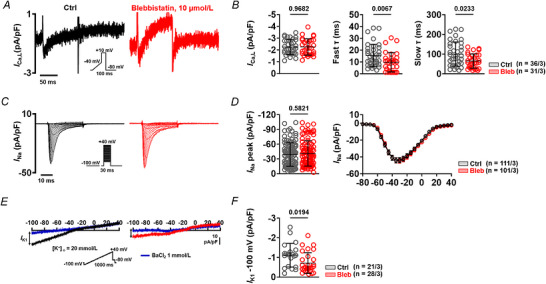
Automated patch‐clamp screening of ion channel function in induced pluripotent stem cell‐derived atrial cardiomyocytes *A*, representative *I*_Ca,L_ traces in the presence and absence of blebbistatin (Bleb, 10 µmol/l). Inset, voltage protocol. *B*, quantification (mean ± SD) of peak *I*_Ca,L_ amplitude and biphasic (fast and slow) inactivation kinetics of *I*_Ca,L_ at +10 mV. *C*, representative *I*_Na_ current traces in the presence and absence of blebbistatin (Bleb, 10 µmol/l). Inset, voltage protocol with +5 mV increments. *D*, peak *I*_Na_ amplitude and *I*–*V* curve (both mean ± SD). *E*, representative traces showing basal inward rectifier current (*I*_K1_) in the presence of 20 mmol/l KCl (black/red) and BaCl_2_ (blue). Inset, voltage protocol. *F*, quantification (mean ± SD) of peak *I*_K1_ amplitude at −100 mV. Comparisons were made using Student's unpaired *t* test or the Mann–Whitney *U* test *versus* Ctrl. *P*‐values *versus* Ctrl are indicated. *n*/*N* = induced pluripotent stem cell‐derived atrial cardiomyocytes/batches. Abbreviations: Bleb, blebbistatin; Ctrl, control.

Ion channel screening was performed using an automated patch‐clamp system, and no difference in *I*_Na_ or *I*_Ca,L_ peak densities were observed between control and blebbistatin‐treated groups (Fig. 5*A*–*D*). Interestingly, when biphasic inactivation kinetics of *I*_Ca,L_ at +10 mV were estimated, the fast phase [thought to be dominated by Ca^2+^‐dependent inactivation (CDI)], in addition to the slow phase (during which voltage‐dependent inactivation is important) were both found to be significantly quicker in the blebbistatin‐treated group (Fig. 5*B*).

In addition, basal *I*_K1_ density was examined using a previously reported ramp protocol and a high‐extracellular‐K^+^ (20 mmol/l KCl) solution, followed by blocking the current with BaCl_2_ (1 mmol/l) (Fig. 5*E*). The *I*_K1_ was analysed as Ba^2+^‐sensitive current at −100 mV. Blebbistatin‐treated iPSC‐aCM showed significantly lower *I*_K1_ density compared with control cells (Fig. 5*E* and *F*).

Taken together, these findings demonstrate that reduced Ca^2+^ buffering leads to elevated diastolic Ca^2+^ levels, increased SR Ca^2+^ leak, altered *I*_Ca,L_ inactivation kinetics and reduced *I*_K1_ density.

### Ca^2+^ chelation restores ion channel function in blebbistatin‐treated iPSC‐aCM

To determine whether Ca^2+^ chelation could mitigate blebbistatin effects and to address the possibility of direct effects of blebbistatin on *I*_Ca,L_, EGTA (10 mmol/l) was added to the internal solution in automated patch‐clamp experiments. Ion channel screening was performed using the automated patch‐clamsystem, in which *I*_Na_, *I*_Ca,L_ and *I*_K1_ were measured in the same cells in sequential order, as described above.

**Figure 6:**
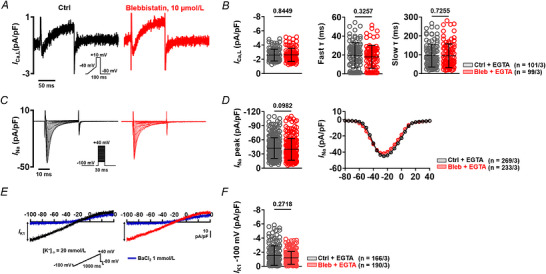
Automated patch‐clamp screening of ion channel function in induced pluripotent stem cell‐derived atrial cardiomyocytes using internal solution containing the Ca^2+^ chelator EGTA *A*, representative *I*_Ca,L_ traces in the presence and absence of blebbistatin (Bleb, 10 µmol/l). Inset, voltage protocol. *B*, quantification (mean ± SD) of peak *I*_Ca,L_ amplitude and biphasic (fast and slow) inactivation kinetics of *I*_Ca,L_ at +10 mV. *C*, representative *I*_Na_ current traces in the presence and absence of blebbistatin (Bleb, 10 µmol/l). Inset, voltage protocol with +5 mV increments. *D*, peak *I*_Na_ amplitude and *I*–*V* curve (both mean ± SD). *E*, representative traces showing basal inward rectifier current (*I*_K1_) in the presence of 20 mmol/l KCl (black/red) and BaCl_2_ (blue). Inset, voltage protocol. *F*, quantification (mean ± SD) of peak *I*_K1_ amplitude at −100 mV. Comparisons were made using Student's unpaired *t* test or the Mann–Whitney *U* test *versus* Ctrl. *P*‐values *versus* Ctrl are indicated. *n*/*N* = induced pluripotent stem cell‐derived atrial cardiomyocytes/batches. Abbreviations: Bleb, blebbistatin; Ctrl, control; EGTA, ethylene glycol tetraacetic acid.

In the presence of EGTA, *I*_Na_, *I*_Ca,L_ and *I*_K1_ current densities were not significantly different between control and blebbistatin‐treated iPSC‐aCM (Fig. 6). Furthermore, both components of biphasic inactivation kinetics of *I*_Ca,L_ were comparable between the groups (Fig. 6*B*). These findings suggest that compensating for reduced Ca^2+^ buffering might reverse blebbistatin‐induced changes. Furthermore, direct effects of blebbistatin on *I*_Ca,L_ can also be excluded, based on these results.

## Discussion

In this study, we showed that blebbistatin, a myosin II ATPase inhibitor, selectively impairs Ca^2+^ buffering in human iPSC‐aCM, thereby inducing potential pro‐arrhythmic changes. This effect is mediated primarily by blebbistatin‐induced reduction in affinity of intracellular Ca^2+^ buffers (myofilaments), leading to elevated diastolic Ca^2+^ levels and increased SR Ca^2+^ leak. Blebbistatin did not alter SR Ca^2+^ release or uptake during steady‐state systolic pacing. Interestingly, although blebbistatin did not significantly impact peak density of *I*_Na_ or *I*_Ca,L_, inactivation kinetics of *I*_Ca,L_ were altered. Furthermore, *I*_K1_ density was significantly reduced after blebbistatin. Ca^2+^ chelation with EGTA normalised *I*_K1_ ion channel function and *I*_Ca,L_ inactivation kinetics. These findings, for the first time, provide mechanistic insight into how blebbistatin disrupts Ca^2+^ homeostasis, leading to pro‐arrhythmic changes in cardiomyocytes.

### Mechanistic insights into the effects of blebbistatin on Ca^2+^ handling

Blebbistatin is a small molecule inhibitor with high affinity and selectivity for myosin II ATPase (Allingham et al., 2005; Farman et al., 2008). It has been reported that blebbistatin is a safe EC uncoupler (Fedorov et al., 2007), and it is widely used in cardiac optical mapping research. However, there is also evidence that blebbistatin might, in fact, significantly prolong action potential duration, leading to altered ventricular fibrillation initiation threshold (Brack et al., 2013). Recently, we used blebbistatin to modulate Ca^2+^ sensitivity indirectly in Langendorff‐perfused mouse hearts, which, in turn, significantly increased arrhythmia inducibility (Fakuade et al., 2024). Likewise, a reduction in Ca^2+^ sensitivity was achieved in a concentration‐dependent manner in mouse cardiac muscle preparations (Baudenbacher et al., 2008; Dou et al., 2007). However, such studies are mainly based on quantification of the effects of blebbistatin on contractile force and do not directly quantify effects on cytosolic Ca^2+^ buffering and Ca^2+^ homeostasis.

In the present study, we quantified the effect of blebbistatin on Ca^2+^ handling and showed that Ca^2+^ desensitisation is promoted via reduced affinity of intracellular Ca^2+^ buffers, as evidenced by the increased buffering dissociation constant (*K*_d_), without changes in the maximum buffering capacity (*B*_max_). Notably, the endogenous Ca^2+^ buffer capacity typically accounts for 98–99% of cytosolic Ca^2+^, leaving only 1–2% free (Neher & Augustine, 1992; Smith & Eisner, 2019), thus even modest alterations in buffering might significantly alter free Ca^2+^ dynamics. In our experiments, blebbistatin caused altered diastolic and systolic Ca^2+^ levels, with subsequent increase of SR Ca^2+^ leak, demonstrated by increased incidence of SCaEs, which is well known to be pro‐arrhythmic (Hove‐Madsen et al., 2004; Neef et al., 2010; Voigt et al., 2012). Interestingly, although blebbistatin caused a reduction in Ca^2+^ buffering, SERCA and NCX activity were not affected. This finding might suggest that Ca^2+^ buffering in cardiomyocytes is compartmentalised (Eisner et al., 2023). The lack of impact of blebbistatin on *I*_Ca,L_ and *I*_Na_ peak density further highlights the specific effect of blebbistatin on Ca^2+^ buffer desensitisation, thereby affecting downstream intracellular Ca^2+^ handling.

### Pro‐arrhythmic implications

The observed effects of blebbistatin in iPSC‐aCM have significant pro‐arrhythmic implications. We showed that blebbistatin causes an elevated diastolic Ca^2+^ level and increases SR Ca^2+^ leak, both of which are well‐established triggers of delayed after‐depolarisations, which can lead to cardiac arrhythmia (Chelu et al., 2009; Kujala et al., 2012; Priori & Corr, 1990; Shan et al., 2012). In the present study, we also observed reduced *I*_K1_ density after blebbistatin. A direct channel‐blocking effect of blebbistatin is unlikely, owing to the fact that these changes were mitigated after Ca^2+^ chelation with EGTA. This is in line with the primary physiological property of the *I*_K1_ channel (i.e. rectification), which is driven by the intracellular concentrations of bivalent cations (Mg^2+^ and Ca^2+^) and polyamines (Lopatin et al., 1994; Matsuda et al., 1987; Mazzanti & Difrancesco, 1989; Zaza et al., 1998). Thus, elevated free Ca^2+^ following blebbistatin application might alter *I*_K1_ kinetics. In a simulation study, lower *I*_K1_ density was associated with significantly prolonged repolarisation phase of the action potential and depolarisation of the resting membrane potential, causing increased incidence of early after‐depolarisations and delayed after‐depolarisations (Sung et al., 2006). Additionally, we observed that increased diastolic Ca^2+^ led to enhanced inactivation kinetics of *I*_Ca,L_, which is in line with increased free cytosolic Ca^2+^. The processes of CDI and voltage‐dependent inactivation are interdependent and therefore we hypothesise that alterations in CDI occurring upon blebbistatin treatment might drive changes in voltage‐dependent inactivation. The fact that EGTA, a slow buffer, did not completely abolish CDI in the presence or absence of blebbistatin, suggests that Ca^2+^ entering via *I*_Ca,L_ can exert CDI and that this is not affected by blebbistatin, pointing towards compartmentalisation of Ca^2+^ signalling. Altered channel kinetics could potentially affect action potential duration and thereby increase pro‐arrhythmic activity. Interestingly, there is a study reporting that peak *I*_Ca,L_ and its inactivation constants remain unaltered in the presence of blebbistatin (Dou et al., 2007). However, it is worth noting that the presence of EGTA in the pipette solution in that study could potentially mitigate the effects of blebbistatin, as was the case with EGTA in our study (Fig. 6*B*).

Taken together, we have shown that blebbistatin might be able to promote a multifaceted pro‐arrhythmic substrate, which is likely to be driven by altered Ca^2+^ buffering. Future studies are required to investigate the relative contributions of the observed effects, particularly the interplay between impaired Ca^2+^ buffering and Ca^2+^‐dependent ion channels.

### Therapeutic outlook

Our findings suggest that the effects of blebbistatin might be reversed through additional Ca^2+^ chelation using EGTA. Likewise, the application of exogenous EGTA as a Ca^2+^ buffer has been shown to prevent the propagation of arrhythmogenic Ca^2+^ waves (MacQuaide et al., 2010), and recently we showed, in a model of reduced intracellular Ca^2+^ buffering, that a higher incidence of SCaEs could be rescued using the Ca^2+^ sensitiser EMD57033 (Fakuade et al., 2024). These insights suggest potential new therapeutic approaches centred around the modulation of intracellular Ca^2+^ buffering properties, such as altering Ca^2+^ sensitivity of Ca^2+^‐binding proteins (e.g. cardiac troponin C), or the development of compounds that directly alter Ca^2+^ buffering. The above‐mentioned approaches hold potential benefit for management of AF, because currently available treatment strategies are based on ion channel blockade, often failing to address underlying Ca^2+^‐handling‐specific mechanisms (Joglar et al., 2024; Liutkute et al., 2022).

### Potential limitations

This study demonstrates, for the first time, that the reduction in Ca^2+^ buffering is a primary mechanistic driver of the pro‐arrhythmic substrate induced by blebbistatin. Some limitations, however, should be highlighted. First, the use of human iPSC‐aCM as a model system, although powerful, does not fully recapitulate the properties of adult human cardiomyocytes, owing to issues related to cell maturity (Itzhaki, Maizels et al., 2011; Itzhaki, Rapoport et al., 2011; Kim et al., 2015; van den Berg et al., 2015). Nevertheless, within this project we used iPSC‐aCM at an appropriate post‐differentiation age to minimise these limitations and enhance the relevance of the findings (Hwang et al., 2015; Seibertz, Sutanto et al., 2023). We assume that our findings in atrial iPSC‐CM are likely to apply also to ventricular iPSC‐CM, although this has to be tested in future studies.

Second, our experiments were conducted in *in vitro* conditions; thus, complex three‐dimensional cell‐to‐cell interactions within a tissue format might have been overlooked. However, as discussed, evidence has been demonstrated in our laboratory on the effects of blebbistatin at the tissue level, in Langendorff‐perfused mouse hearts (Fakuade et al., 2024). Importantly, it was observed in that study that atrial refractory periods, measured at each experimental step and across various K^+^ concentrations, remained comparable to control conditions. This finding highlights that the observed changes were likely to be driven by altered Ca^2+^ buffering. We acknowledge that there are differences in the absolute values of *I*_Ca,L_ densities measured in manual and automated patch‐clamp experiments. We attribute this to variation in the spatial configuration of the cell in the given technique, rather than intrinsic physiological variability. In manual patch clamp, cells adhere to glass coverslips, whereas automated patch clamp requires cellular suspension. Importantly, pharmacological response in both the techniques was similar, supporting the validity of the findings.

A third potential limitation in the present study concerns the contribution of SK channels, which have gained much attention in the literature recently. These channels, however, are thought to be more important in the setting of AF (Heijman et al., 2023), whereas our study uses healthy iPSC‐CM. Although we identified significant reductions in Ca^2+^ buffering and changes in membrane currents, the present study did not investigate downstream molecular effects of altered levels of free cytosolic Ca^2+^, such as signalling pathways mediated by CaMKII (Maier & Bers, 2007) and altered gene transcription (Rinne et al., 2010). In addition, it has been reported that EC uncoupling might cause a shift in cellular metabolic state (Bode et al., 2024; Kuzmiak‐Glancy et al., 2015), which, in turn, might alter ATP‐dependent current, such as *I*_K,ATP_ (Garrott et al., 2017; Kane et al., 2005). Clearly, future work is required to explore mechanisms underlying the pro‐arrhythmic substrate caused by reduced cytosolic Ca^2+^ buffering.

### Conclusions

In this study, we demonstrated that blebbistatin, a myosin II ATPase inhibitor, impairs Ca^2+^ buffering by reducing the affinity of intracellular Ca^2+^ buffers, resulting in elevated diastolic Ca^2+^ levels, leading to increased SR Ca^2+^ leak, reduced *I*_K1_ density and enhanced *I*_Ca,L_ inactivation. Our findings align with previous studies in which investigators reported pro‐arrhythmic potential of blebbistatin (Brack et al., 2013; Kappadan et al., 2020). Despite its widespread use in cardiac optical mapping research as a safe EC uncoupler (Dou et al., 2007; Fedorov et al., 2007), our data suggest that blebbistatin might introduce unintended pro‐arrhythmic activity by disrupting Ca^2+^ dynamics. Importantly, our findings support the use of blebbistatin incubation as a research model to mimic reduced Ca^2+^ buffering.

## Additional information

## Competing interests

None declared.

## Author contributions

I.S. and N.V. designed the studies. I.S., K.R., M.G., L.B., A.L., F.S., F.E.F., F.E.M. and N.V. performed the research and analysed the data. K.R., L.B. and A.L. provided cellular material. I.S., F.E.M. and N.V. wrote the manuscript. All authors gave consent for the publication of the manuscript and agree to be accountable for all aspects of the work in ensuring that questions related to the accuracy or integrity of any part of the work are appropriately investigated and resolved. All persons designated as authors qualify for authorship, and all those who qualify for authorship are listed.

## Funding

This work was supported by grants from the Deutsche Forschungsgemeinschaft (DFG) to N.V. (VO1568/3‐1, VO1568/3‐2, VO1568/4‐1, VO1568/6‐1, SFB1002 project A13 and under Germany's Excellence Strategy, EXC 2067/1, 390729940) to N.V. and from the German Center for Cardiovascular Research to N.V. (DZHK 81X4300102, 81X4300133, 81X4300136, 81X4300135, 81X4300140). This work was also supported by a scholarship from the German Academic Exchange Service (DAAD) to I.S. and the Kaltenbach‐Doktorandenstipendium from the Deutsche Herzstiftung e.V. to K.R.

## Supporting information


Peer Review History

## Data Availability

The data that support the findings of this study are available from the corresponding author upon reasonable request.
