# Supplementary material for: Blebbistatin reduces calcium buffering in cardiomyocytes: Consequences for cellular electrophysiology
Source: J Physiol. 2025 Aug 25;604(16):6858–71. doi: 10.1113/JP287545 (PMC13481002; doi:10.1113/JP287545)
Supplement: Supplementary file 1 [file TJP-604-6858-s001.pdf]

The Journal of Physiology

Blebbistatin reduces calcium buffering in cardiomyoctes: Consequences for cellular electrophysiology

Izzatullo Sobitov, Katharina Ritzenhoff, Marie Gaulrapp, Lea Becker, Aiste Liutkute, Fitzwilliam Seibertz, Funsho E. Fakuade, Fleur E. Mason, and Niels Voigt
DOI: 10.1113/JP287545

Corresponding author(s): Niels Voigt (niels.voigt@med.uni-goettingen.de)

The referees have opted to remain anonymous.

Review Timeline:

Submission Date:	31-Jan-2025
Editorial Decision:	04-Mar-2025
Revision Received:	02-Jun-2025
Accepted:	01-Jul-2025

Senior Editor: Bjorn Knollmann

Reviewing Editor: Brian Delisle

Transaction Report:

(Note: With the exception of the correction of typographical or spelling errors that could be a source of ambiguity, letters and reports are not edited. Depending on transfer agreements, referee reports obtained elsewhere may or may not be included in this compilation. Referee reports are anonymous unless the Referee chooses to sign their reports.)

Dear Dr Voigt,

Re: JP-RP-2025-287545 "Blebbistatin reduces calcium buffering in cardiomyoctes: Consequences for cellular electrophysiology" by Izzatullo Sobitov, Katharina Ritzenhoff, Marie Gaulrapp, Lea Becker, Aiste Liutkute, Fitzwilliam Seibertz, Fleur E. Mason, Funsho E. Fakuade, and Niels Voigt

Thank you for submitting your manuscript to The Journal of Physiology. It has been assessed by a Reviewing Editor and by 2 expert referees and we are pleased to tell you that it is potentially acceptable for publication following satisfactory major revision.

Please advise your co-authors of this decision as soon as possible.

The referee reports are copied at the end of this email.

Please address all the points raised and incorporate all requested revisions or explain in your Response to Referees why a change has not been made. We hope you will find the comments helpful and that you will be able to return your revised manuscript within 2 months. If you require longer than this, please contact journal staff: jp@physoc.org. Please note that this letter does not constitute a guarantee for acceptance of your revised manuscript.

Your revised manuscript should be submitted online using the link in your Author Tasks: Link Not Available. This link is accessible via your account as Corresponding Author; it is not available to your co-authors. If this presents a problem, please contact journal staff (jp@physoc.org). Image files from the previous version are retained on the system. Please ensure you replace or remove any files that are being revised.

If you do not wish to submit a revised version of your manuscript, you must inform our journal staff (jp@physoc.org) or reply to this email to request withdrawal. Please note that a manuscript must be formally withdrawn from the peer review process at one journal before it may be submitted to another journal.

TRANSPARENT PEER REVIEW POLICY: To improve the transparency of its peer review process, The Journal of Physiology publishes online as supporting information the peer review history of all articles accepted for publication. Readers will have access to decision letters, including Editors' comments and referee reports, for each version of the manuscript, as well as any author responses to peer review comments. Referees can decide whether or not they wish to be named on the peer review history document.

ABSTRACT FIGURES: Authors are expected to use The Journal's premium BioRender account to create/redraw their Abstract Figures. Information on how to access this account is here: https://physoc.onlinelibrary.wiley.com/journal/14697793/biorender-access.

This will enable Authors to create and download high-resolution figures. If authors have used the free BioRender service, they can use the instructions provided in the link above to download a high-resolution version suitable for publication.

The link provided should only be used for the purposes of this submission. Authors will be charged for figures created on this account if they are not related to this manuscript submission.

LANGUAGE EDITING AND SUPPORT FOR PUBLICATION: If you would like help with English language editing, or other article preparation support, Wiley Editing Services offers expert help, including English Language Editing, as well as translation, manuscript formatting, and figure formatting at www.wileyauthors.com/eeo/preparation. You can also find resources for Preparing Your Article for general guidance about writing and preparing your manuscript at www.wileyauthors.com/eeo/prepresources.

RIGOUR AND REPRODUCIBILITY: We recommend authors consult The Journal's Rigour and Reproducibility requirements to ensure that their manuscript is suitable for publication: https://physoc.onlinelibrary.wiley.com/pb-assets/hub-assets/physoc/documents/TJP-Rigour-and-Reproducibility-Requirements.pdf

REVISION CHECKLIST:

Check that your Methods section conforms to journal policy: https://jp.msubmit.net/cgi-bin/main.plex?form_type=display_requirements#methods.

Check that data presented conforms to the statistics policy: https://jp.msubmit.net/cgi-bin/main.plex?form_type=display_requirements#statistics.

Upload a full Response to Referees file. To create your 'Response to Referees': copy all the reports, including any comments from the Senior and Reviewing Editors, into a Microsoft Word, or similar, file and respond to each point, using font or background colour to distinguish comments and responses and upload as the required file type.

Please upload two versions of your manuscript text: one with all relevant changes highlighted and one clean version with no changes tracked. The manuscript file should include all tables and figure legends, but each figure/graph should be uploaded as separate, high-resolution files.

You may also upload:

- 'Potential Cover Art' for consideration as the issue's cover image
- Appropriate Supporting Information (Video, audio or data set: see https://jp.msubmit.net/cgi-bin/main.plex?form_type=display_requirements#supp).

We look forward to receiving your revised submission.

If you have any queries, please reply to this email and we will be pleased to advise.

Yours sincerely,

Bjorn Knollmann
Senior Editor
The Journal of Physiology

REQUIRED ITEMS

- Author photo and profile. First or joint first authors are asked to provide a short biography (no more than 100 words for one author or 150 words in total for joint first authors) and a portrait photograph. These should be uploaded and clearly labelled together in a Word document with the revised version of the manuscript. See Information for Authors for further details.

- The contact information for the person responsible for 'Research Governance' at your institution needs to be provided. This includes their name and an institutional email address. Please ensure the contact is not an author on this paper and provide an alternate contact if necessary, or confirm in the submission form that the author whose email was provided has sole responsibility for research governance. This is the person who is responsible for regulations, principles and standards of good practice in research carried out at the institution, for instance the ethical treatment of animals, the keeping of proper experimental records or the reporting of results.

- You must start the Methods section with a paragraph headed Ethical Approval. If experiments were conducted on humans, confirmation that informed consent was obtained, preferably in writing, that the studies conformed to the standards set by the latest revision of the Declaration of Helsinki and that the procedures were approved by a properly constituted ethics committee, which should be named, must be included in the article file. If the research study was registered (clause 35 of the Declaration of Helsinki), the registration database should be indicated, otherwise the lack of registration should be noted as an exception (e.g. The study conformed to the standards set by the Declaration of Helsinki, except for registration in a database). For further information see: https://physoc.onlinelibrary.wiley.com/hub/human-experiments.

- Please upload separate high-quality figure files via the submission form.

- Please ensure that any tables are editable and in Word format, and wherever possible, embedded in the article file itself.

- Please ensure that the Article File you upload is a Word file.

- Your paper contains Supporting Information of a type that we no longer publish, including supplementary tables and figures. Any information essential to an understanding of the paper must be included as part of the main manuscript and figures. The only Supporting Information that we publish are video and audio, 3D structures, program codes and large data files. Your revised paper will be returned to you if it does not adhere to our Supporting Information Guidelines.

- Papers must comply with the Statistics Policy: https://jp.msubmit.net/cgi-bin/main.plex?form_type=display_requirements#statistics.

In summary:

- If n {less than or equal to} 30, all data points must be plotted in the figure in a way that reveals their range and distribution. A bar graph with data points overlaid, a box and whisker plot or a violin plot (preferably with data points included) are acceptable formats.

- If n > 30, then the entire raw dataset must be made available either as supporting information, or hosted on a not-for-profit repository, e.g. FigShare, with access details provided in the manuscript.

- 'n' clearly defined (e.g. x cells from y slices in z animals) in the Methods. Authors should be mindful of pseudoreplication.

- All relevant 'n' values must be clearly stated in the main text, figures and tables.

- The most appropriate summary statistic (e.g. mean or median and standard deviation) must be used. Standard Error of the Mean (SEM) alone is not permitted.

- Exact p values must be stated. Authors must not use 'greater than' or 'less than'. Exact p values must be stated to three significant figures even when 'no statistical significance' is claimed.

- Please include an Abstract Figure file, as well as the Figure Legend text within the main article file. The Abstract Figure is a piece of artwork designed to give readers an immediate understanding of the research and should summarise the main conclusions. If possible, the image should be easily 'readable' from left to right or top to bottom. It should show the physiological relevance of the manuscript so readers can assess the importance and content of its findings. Abstract Figures should not merely recapitulate other figures in the manuscript. Please try to keep the diagram as simple as possible and without superfluous information that may distract from the main conclusion(s). Abstract Figures must be provided by authors no later than the revised manuscript stage and should be uploaded as a separate file during online submission labelled as File Type 'Abstract Figure'. Please also ensure that you include the figure legend in the main article file. All Abstract Figures should be created using BioRender. Authors should use The Journal's premium BioRender account to export high-resolution images. Details on how to use and access the premium account are included as part of this email.

EDITOR COMMENTS

Reviewing Editor:

Comments for Authors to ensure the paper complies with the Statistics Policy (Required):
Please revise the manuscript to conform to the policies outlined in: https://jp.msubmit.net/cgi-bin/main.plex?form_type=display_requirements#statistics. This included exact p-values and the use of SD (not SEM).

Comments to the Author (Required):
Two expert referees in the field evaluated the manuscript. While the referees thought the work was sound, they expressed minor and major concerns that the manuscript is primarily confirmatory, could benefit from additional experimental studies, and does not significantly impact the field in its current form. Several clarifications and experiments are suggested to improve the significance of the study.

Please also see 'Required Items' above.

Senior Editor:

Comments for Authors to ensure the paper complies with the Statistics Policy (Required):
Please comply with the statistics policy (i.e., SD not SEM)

Comments to the Author:
The work is interesting, but a responsive revision would require addressing the concerns raised in the review, which will require new experiments. The authors also need to better explain the novelty of the work, since the effect of blebbistatin on

buffering has been reported before, and address why pro-arrhythmic effects are postulated whereas antiarrhythmic effects of blebbistatin have been reported in hypertrophic cardiomyopathy models (as pointed out by reviewer 1).

REFEREE COMMENTS

Referee #1:

GENERAL COMMENTS

Sobitov and colleagues present a manuscript examining the impact of blebbistatin on Ca2+ buffering in cardiomyocytes. Blebbistatin is used as an uncoupling agent for excitation-contraction coupling by inhibiting myosinII ATPase often in experimental studies performing optical mapping. The authors simultaneously measured whole-cell NCX currents and intracellular Ca2+ to make this assessment following rapid application of caffeine. They also measure a number of ionic currents. Their findings suggest that blebbistatin reduces Ca2+ buffering myocytes by shifting the buffer dissociation constant consistent with a decrease in affinity for Ca2+. In addition, they observed accelerated inactivation of ICa,L and a reduction in IK1 in blebbistatin treated cells. Overall, they conclude that blebbistatin reduces Ca2+ buffering which impacts the cell physiology in a number of ways which can be proarrhythmic, and this needs to be consider in studies using these agents. Overall, the work has several strengths: the detailed single cell studies using a range of techniques and combining patch clamp measures with intracellular Ca2+ measurements, larger sample sizes are provides for the relatively noisy iPSC-aCM model, and the results are clearly present. However, there are some limitations to the work as currently presented:

1. The finding that blebbistatin reduces calcium sensitivity of the myofilaments and hence calcium buffering in cardiomyocytes has been demonstrated and known for some time with earlier papers by Dou et al., 2006 (PMID: 17615158) and Baudenbacher et al., 2008 (PMID: 19033660). Thus, the findings in the present study seem largely confirmatory. The authors need to better clarify any novel or contradictory findings in this study relative to the literature.

2. Why do the authors use Fluo-3 AM rather than the salt form since they are using a patch pipette. Otherwise, the Flou-3 in cell will be diluted during experiment. Also the salt form may overcome some limitations in compartmentalization of the dye allowing focus on cytosolic compartment.

3. Line 90 states that for whole-cell voltage clamp experiments, all measurements were conducted at room temperature which is a limitation for understanding the impact at physiological temperatures given that many of the proteins and binding show remarkable and differential temperature sensitivity. However, it is confusing as line 65 suggests ICa,L measurements were carried out at 37 C.

4. What is the impact of blebbistatin on the decay of ICa,L shown in Figure 1A? This is a consistency check for the ICa,L measures by automated patch clamp.

5. The authors ICa,L measurements in Figures 5 and 6 examining the impact of blebbistatin in the presence and absence of 10 mm EGTA buffering are more complex to interpret than suggested. This amount of EGTA (10 mM) in the pipette buffers cellular Ca2+ to a level that SR stores are largely depleted and Ca2+ transients do not occur in response to ICa,L currents (no contractions). In contrast, the minimal Ca2+ buffering used in Figure 5 will potentially allow Ca2+-induced Ca2+ transients. Thus, it may be that the impact observed is due to the presence or absence of Ca2+ transients rather than a simple change in buffering of intracellular Ca2+ to a different level.

6. The authors suggest that blebbistatin is pro-arrhythmic, but this contrasts the conclusion from the Knollmann group suggesting blebbistatin in the presence of hypertrophic cardiomyopathy mutations is antiarrhythmic (Baudenbacher et al., 2008, PMID: 19033660). How do the authors reconcile these apparently different results. Is this an atrial vs. ventricular difference?

MINOR COMMENTS

1. The authors state that the biexponential decay of ICa,L can be simply separated into CDI (fast tau) and VDI (slow tau). Unfortunately, while those process tend to segregate in that manner, detail biophysical studies suggest there is clear overlap. Perhaps the authors should be more circumspect in this description.

Referee #2:

The study by Sobitov et al. investigates the excitation-contraction uncoupling effects of blebbistatin on Ca2+ signaling properties of human induced pluripotent stem-cell derived atrial cardiomyocytes. The study provides evidence that reduced intracellular Ca2+ buffering by blebbistatin increases the diastolic Ca2+ transients, SR Ca2+ leak and spark frequency, increases ICaL inactivation, without affecting the SR Ca2+ content and ICaL amplitude. These changes the cellular electrophysiological dynamics, authors suggest, are responsible for reported proarrhythmic effects of blebbistatin. Although the manuscript is well-written, the authors fail to provide evidence for some of their assertions or explain the contradictions in their results. The MS will be greatly improved by providing more details on methods and results sections.

Major concerns:

1. How did the authors confirm that their derived cardiomyocytes indeed were atrial of nature? Did the Authors perform any action potential measurements, or did they test the expression of subtype-specific cardiac markers like MLC2a? Did the author test the effects of acute application of Retinoic acid on the calcium signaling parameters? The MS would be greatly strengthened by establishing the atrial nature of these cells.

2. There is no indication on how SR Ca2+ leak was measured. Was it measured by the application of tetracaine in zero calcium zero sodium solutions? Was the leak then quantified as the fraction of caffeine-induced release? Please clarify and include the description in the Methods section. Why was 10mM caffeine used? Generally 3-5 mM caffeine produce the maximum calcium release from SR in cardiomyocytes. Use of the higher concentrations may activate calcium entry on hemi-channels, reported by some investigators.

3. Fig.1 shows ICaL current around 20 pA/pF, while Figs.5-6 show only ICaL amplitudes of 2-5 pA/pF. How can the authors explain these large difference in their results? Are the cells with larger calcium current autophosphrylated? The larger ICa density cells are more consistent with those reported for hiPSC-CMs in the literature! Did the measured Ca-transients reflect this large difference in Ica?

Figure 2 shows greater dispersion of INCX in blebistatine treated cells consistent with larger Ca-transients. Why would increasing the calcium buffering result in larger Ca-transients? Does the dispersion arise from variance in Ica in different cells?

4. Small-conductance Ca2+ activated potassium channels were shown to be present in both ventricles and atria, however functional activation of SK channels is significantly more prominent in atria. Increasing number of studies show the inhibition of SK channels as a potential antiarrhythmic treatment option in AF and other atrial arrhythmias. Did the Authors examine the effect of reduced Ca2+ buffering on the Ca2+ dependent K+ current in their atrial specific CMs. I would recommend doing such experiments.

5. The Authors have used blebbistatin in the concentration of 10 µM. Did the Authors test its dose-dependent effects on the intracellular Ca2+ signaling properties of hiPSC derived atrial CMs?

6. iPSC derived cardiomyocytes have the advantage that they show spontaneous automaticity. Were these cells able to maintain spontaneous beating? If so, did the faster calcium dependent inactivation and the reduced IK1 result in faster beating rates?

7. Authors claim that the intracellular Ca2+ changes due to blebbistatin results in pro-arrhythmic changes. Did the Authors measure any indicators of arrhythmia for instance, EADs or DADs in these cells?

8. Did the authors check on calcium channel current kinetics and amplitude when Ba2+ was the charge carrier through the channel. This may provide some insight on the buffering issue, because acute application of Ba2+ containg solution lasting only a few seconds that fails to activate CICR and rise of cytosolic calcium transients may shed some light on the authors' proposal of compartmented calcium pools.

Minor:

Starting almost every sentence with mean {plus minus} SEM in the figure legends is somewhat odd. I would recommend saying for example Quantification of CaT amplitude instead of Mean {plus minus} SEM CaT amplitude etc.

END OF COMMENTS

To-do-List & Required Items

- 'Potential Cover Art' for consideration as the issue's cover image

Author photo and profile.

The contact information for the person responsible for 'Research Governance' at your institution needs to be provided.

Your paper contains Supporting Information of a type that we no longer publish

Statistics Policy

Abstract Figure file, as well as the Figure Legend text

Response to Editor’s comments

We greatly appreciate the thoughtful efforts of the Editors and Reviewers on our manuscript and are thankful for the constructive feedback, which has significantly enhanced our work.

In the revised version, we have updated the format of our submitted documents as requested by the Editorial Office. In particular, we have revised the presentation of statistical analysis.

In addition, in the revised version we highlight the novelty of our work and discuss that both increased or decreased buffering can be arrhythmogenic. We also refer to an excellent editorial by Knollmann and Bers (Knollmann & Bers, 2024) which discusses this and accompanies our previous study (Fakuade *et al.*, 2024).

Page 4, line 35: “Considering that about 99% of intracellular Ca^{2+} is bound to buffers, even minor perturbations in the amount of Ca^{2+} buffered may have a major impact on free Ca^{2+}(Smith & Eisner, 2019), thereby influencing EC coupling. Both increased and reduced buffering capacity can increase arrhythmia susceptibility, as discussed recently in an editorial by Knollmann and Bers (Knollmann & Bers, 2024)”

Page 4, line 40: “In this study, we examine the direct effects of blebbistatin (10 µmol/L). We investigate whether blebbistatin selectively disrupts intracellular Ca^{2+} buffering, thereby inducing pro-arrhythmic changes in Ca^{2+} handling and cellular electrophysiology.”

Below, we have responded to the specific comments from the Reviewers.

Response to comments of Reviewer 1

Sobitov and colleagues present a manuscript examining the impact of blebbistatin on Ca^{2+} buffering in cardiomyocytes. Blebbistatin is used as an uncoupling agent for excitation-contraction coupling by inhibiting myosinII ATPase often in experimental studies performing optical mapping. The authors simultaneously measured whole-cell NCX currents and intracellular Ca^{2+} to make this assessment following rapid application of caffeine. They also measure a number of ionic currents. Their findings suggest that blebbistatin reduces Ca^{2+} buffering myocytes by shifting the buffer dissociation constant consistent with a decrease in affinity for Ca^{2+}. In addition, they observed accelerated inactivation of $I_{Ca,L}$ and a reduction in I_{K1} in blebbistatin treated cells. Overall, they conclude that blebbistatin reduces Ca^{2+} buffering which impacts the cell physiology in a number of ways which can be proarrhythmic, and this needs to be consider in studies using these agents. Overall, the work has several strengths: the detailed single cell studies using a range of techniques and combining patch clamp measures with intracellular Ca^{2+} measurements, larger sample sizes are provides for the relatively noisy iPSC-aCM model, and the results are clearly present. However, there are some limitations to the work as currently presented:

We greatly appreciate the Reviewer's positive feedback and their constructive comments, which have led us to make improvements to the manuscript.

1. The finding that blebbistatin reduces calcium sensitivity of the myofilaments and hence calcium buffering in cardiomyocytes has been demonstrated and known for some time with earlier papers by Dou et al., 2006 (PMID: 17615158) and Baudenbacher et al., 2008 (PMID: 19033660). Thus, the findings in the present study seem largely confirmatory. The authors need to better clarify any novel or contradictory findings in this study relative to the literature.

We acknowledge that the literature cited by the Reviewer indeed present important findings, using readouts such as force of contraction to show the effects of blebbistatin. However, those studies did not *directly* measure effects of blebbistatin on cytosolic Ca^{2+} buffering, including parameters such as B_{max} and K_d. Furthermore, it has been questioned whether force of contraction studies can be used as conclusive evidence of altered Ca^{2+} buffering, as it is not the sole factor governing the relationship between $[Ca^{2+}]_i$ and force, as discussed by Smith and Eisner (Smith & Eisner, 2019). To our knowledge, therefore, our study is the first to directly measure the effects of blebbistatin on cytosolic Ca^{2+} buffering in cardiomyocytes (iPSC-CM).

In order to better clarify this important point, we have cited the two studies highlighted by this reviewer and added the following text in the discussion:

Page 12, line 263: "Similarly, Ca^{2+} sensitivity reduction was achieved in a concentration-dependent manner in mouse cardiac muscle preparations (Dou *et al.*, 2007; Baudenbacher *et al.*, 2008). However, such studies are mainly based on quantification of blebbistatin effects on contractile force and do not directly quantify effects on cytosolic Ca^{2+} buffering and Ca^{2+} homeostasis."

2. Why do the authors use Fluo-3 AM rather than the salt form since they are using a patch pipette. Otherwise, the Fluo-3 in cell will be diluted during experiment. Also the salt form may overcome some limitations in compartmentalization of the dye allowing focus on cytosolic compartment.

We apologise for any confusion. The experiments involving simultaneous patch-clamp and epifluorescent measurements were performed with fluo-3 loaded cells as well as pipette solution containing fluo-3 pentapotassium salt, originally described in the Supplements. We have now added this information to the Methods section of the revised manuscript for clarity:

Page 6, line 89: "The pipette solution (in mmol/L: EGTA 0.02; GTP-Tris 0.1; HEPES 10; K-aspartate 92; KCl 48; MgATP 1; Na_2ATP 4; pH = 7.2 adjusted with KOH) contained fluo-3 pentapotassium salt, 0.1 mmol/L (Thermo Scientific)."

3. Line 90 states that for whole-cell voltage clamp experiments, all measurements were conducted at room temperature which is a limitation for understanding the impact at physiological temperatures given that many of the proteins and binding show remarkable and differential temperature sensitivity. However, it is confusing as line 65 suggests ICa,L measurements were carried out at 37 C.

We thank the Reviewer for this comment. Whereas manual patch-clamp experiments were performed at 37 °C, all experiments performed by automated patch-clamp (APC) were carried out at room temperature in order to enable calcium- and fluoride-assisted seal formation (Milligan *et al.*, 2009; Seibertz *et al.*, 2022), which is less efficient at 37 °C.

We have now extended the following sentence in the Methods section about APC:

Page 8, line 165: "All measurements were conducted at room temperature, allowing calcium- and fluoride-assisted seal formation (Milligan *et al.*, 2009; Seibertz *et al.*, 2022)."

Analysis of ion channel activity in the present study, specifically $I_{Ca,L}$ peak as well as fast and slow inactivation kinetics, showed similar effects of blebbistatin at both room and physiological temperatures (Figure 1). Furthermore, there is evidence that I_{K1} is not temperature-dependent (Voigt *et al.*, 2010).

4. What is the impact of blebbistatin on the decay of ICa,L shown in Figure 1A? This is a consistency check for the ICa,L measures by automated patch clamp.

We appreciate the Reviewer's suggestion and we have performed the analysis accordingly. Please refer to Figure 1. The observed changes in automated patch-clamp experiments were consistent with manual patch-clamp data.

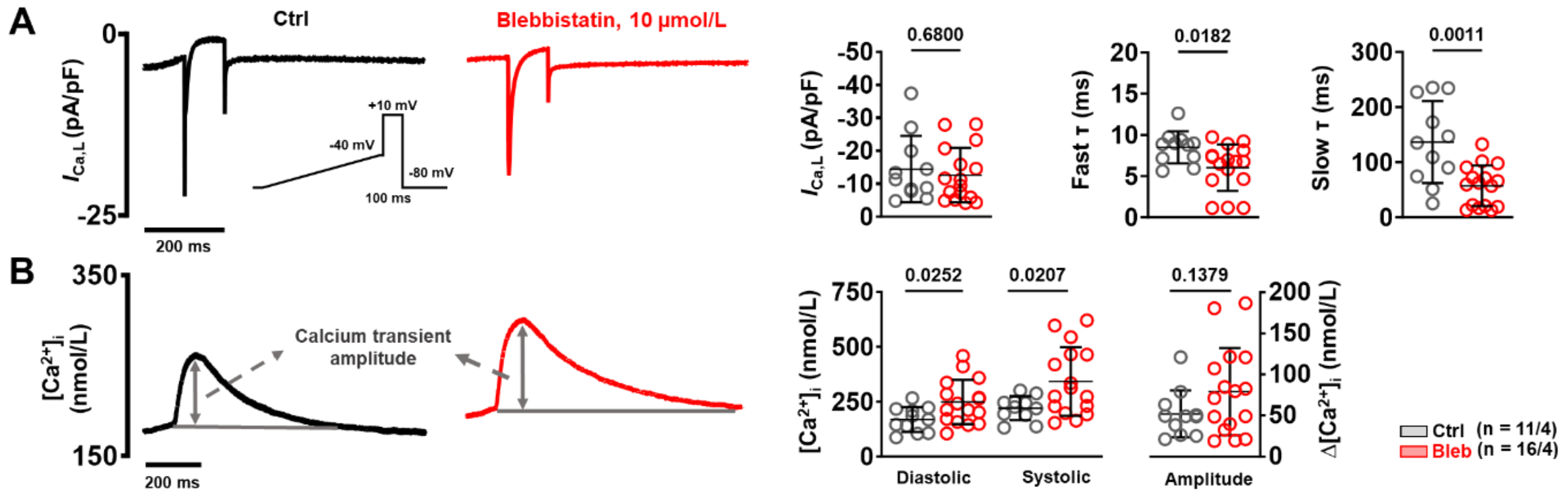

Figure 1. $I_{Ca,L}$ and Ca^{2+} transients (CaT) in iPSC-aCM. **A,** Representative traces of $I_{Ca,L}$ with voltage-clamp protocol (inset) and quantification (mean ± SD) of peak $I_{Ca,L}$ amplitude and inactivation (tau of inactivation) kinetics. **B,** Representative traces of CaT (Fluo-3 AM) and quantification (mean ± SD) of diastolic and systolic $[Ca^{2+}]_i$ and CaT amplitude. Cells were pretreated with blebbistatin (10 µmol/L). Comparisons made using unpaired Student's t-test, Mann Whitney's U-test. p values vs Ctrl. n/N = iPSC-aCMs/batches.

5. The authors $I_{Ca,L}$ measurements in Figures 5 and 6 examining the impact of blebbistatin in the presence and absence of 10 mm EGTA buffering are more complex to interpret than suggested. This amount of EGTA (10 mM) in the pipette buffers cellular Ca2+ to a level that SR stores are largely depleted and Ca2+ transients do not occur in response to ICa,L currents (no contractions). In contrast, the minimal Ca2+ buffering used in Figure 5 will potentially allow Ca2+-induced Ca2+ transients. Thus, it may be that the impact observed is due to the presence or absence of Ca2+ transients rather than a simple change in buffering of intracellular Ca2+ to a different level.

We apologise if there was any confusion concerning the aim of this experiment. We wanted to use EGTA to abolish Ca^{2+}-induced Ca^{2+} release from the SR and therefore cytosolic Ca^{2+} transients, thus allowing us to exclude any direct effect of blebbistatin on Ca^{2+} channel inactivation.

To clarify this, we extended/added the following sentences in the Results section:

Page 11, line 232: "To determine whether Ca^{2+} chelation could mitigate blebbistatin effects, as well as to address the possibility of direct effects of blebbistatin on $I_{Ca,L}$, ethylene glycol tetraacetic acid (EGTA, 10 mmol/L) was added to the internal solution in APC experiments."

and

Page 11, line 241: "Furthermore, direct effects of blebbistatin on $I_{Ca,L}$ can also be excluded, based on these results."

6. The authors suggest that blebbistatin is pro-arrhythmic, but this contrasts the conclusion from the Knollmann group suggesting blebbistatin in the presence of hypertrophic cardiomyopathy mutations is antiarrhythmic (Baudenbacher et al., 2008, PMID: 19033660). How do the authors reconcile these apparently different results. Is this an atrial vs. ventricular difference?

We refer the Reviewer to an excellent editorial by Knollmann and Bers (Knollmann & Bers, 2024), describing the biphasic relationship between Ca^{2+} buffering and arrhythmogenic risk (both too much and too little Ca^{2+} buffering power can increase risk for arrhythmia). Hypertrophic cardiomyopathy has been associated with increased Ca^{2+} buffering (Schober *et al.*, 2012)**.** Therefore, we suggest that normalisation of buffering by a Ca^{2+} desensitiser in this scenario would protect against arrhythmia. On the other hand, reducing buffering to below normal levels, as in the current study, is also thought to be pro-arrhythmic. Indeed, we have recently shown in an *ex vivo* mouse heart model that blebbistatin increases arrhythmia inducibility (Fakuade *et al.*, 2024).

MINOR COMMENT: The authors state that the biexponential decay of $I_{Ca,L}$ can be simply separated into CDI (fast tau) and VDI (slow tau). Unfortunately, while those process tend to segregate in that manner, detail biophysical studies suggest there is clear overlap. Perhaps the authors should be more circumspect in this description.

We thank the Reviewer for highlighting an important nuance in interpreting inactivation kinetics of $I_{Ca,L}$. Although $I_{Ca,L}$ inactivation has broadly been described as involving Ca^{2+}-dependent and voltage-dependent processes with distinct time constants (τ) that can be quantified from bi-exponential current decay, there is evidence showing that these processes are interdependent and that the underlying mechanisms influence each other (Findlay, 2004; Mahajan *et al.*, 2008; Grandi *et al.*, 2010).

Consequently, we have toned-down the wording in the Results section:

Page 10, line 220: "Interestingly, when biphasic inactivation kinetics of $I_{Ca,L}$ at +10 mV were estimated, the fast phase (thought to be dominated by Ca^{2+}- dependent inactivation, CDI), as well as the slow phase (during which VDI is important) were both found to be significantly quicker in the blebbistatin-treated group (Figure 5B)."

We have also changed the legend of figures 5B and 6B, where we no longer specify CDI and VDI.

Further, due to the interdependence of both processes, we suggest that alterations in CDI may drive changes in VDI. The EGTA data are also in line with this hypothesis, as both fast and slow inactivation of $I_{Ca,L}$ showed no difference upon blebbistatin treatment. We altered the text in the Discussion as follows:

Page 13, line: 296: "Additionally, we observed that increased diastolic Ca^{2+} led to enhanced inactivation kinetics of $I_{Ca,L}$, which is in line with increased free cytosolic Ca^{2+}. The processes of CDI and VDI are inter-dependent and therefore we hypothesise that alterations in CDI occurring upon blebbistatin treatment may drive changes in VDI."

Response to comments of Reviewer 2

The study by Sobitov et al. investigates the excitation-contraction uncoupling effects of blebbistatin on Ca^{2+} signaling properties of human induced pluripotent stem-cell derived atrial cardiomyocytes. The study provides evidence that reduced intracellular Ca^{2+} buffering by blebbistatin increases the diastolic Ca^{2+} transients, SR Ca^{2+} leak and spark frequency, increases I_{CaL} inactivation, without affecting the SR Ca^{2+} content and I_{CaL} amplitude. These changes the cellular electrophysiological dynamics, authors suggest, are responsible for reported proarrhythmic effects of blebbistatin. Although the manuscript is well-written, the authors fail to provide evidence for some of their assertions or explain the contradictions in their results. The MS will be greatly improved by providing more details on methods and results sections.

We thank the Reviewer for their feedback and we have made revisions to the manuscript which we believe address the Reviewer's comments and concerns.

1. How did the authors confirm that their derived cardiomyocytes indeed were atrial of nature? Did the Authors perform any action potential measurements, or did they test the expression of subtype-specific cardiac markers like MLC2a? Did the author test the effects of acute application of Retinoic acid on the calcium signaling parameters? The MS would be greatly strengthened by establishing the atrial nature of these cells.

We thank the Reviewer for this comment. While this study focuses on blebbistatin's effect on Ca^{2+} buffering and ion channels using iPSC-derived atrial cardiomyocytes, our group has previously demonstrated and characterised the atrial phenotype of this cell line (Seibertz *et al.*, 2023*a*). It has been shown that the atrial-subtype directed differentiation protocol produces cardiomyocytes that display distinct atrial-specific properties, notably atrial-like electrophysiology. Although, we did not repeat subtype validation experiments in this study, the same established differentiation protocol and cell line were used (Cyganek *et al.*, 2018).

We did not test acute application of retinoic acid on calcium handling as suggested by this reviewer. It is important to note that retinoic acid is applied on day 3 and 5 post-initiation of differentiation, and it is not present in later stages of myocyte maturation. Electrophysiological and calcium handling experiments were performed after day 35-40 in the absence of retinoic acid (Methods section of the revised manuscript). We therefore feel that investigating acute effects of retinoic acid on cellular electrophysiology is beyond the scope of our study.

Furthermore, we hypothesise that the findings in this study likely apply to iPSC-derived ventricular cardiomyocytes as well and future studies will explicitly test this. We have now added text in the Potential Limitations section:

Page 14, line: 233: "We assume that our findings in atrial iPSC-CM are likely to apply also to ventricular iPSC-CM, although this has to be tested in future studies."

2. There is no indication on how SR Ca2+ leak was measured. Was it measured by the application of tetracaine in zero calcium zero sodium solutions? Was the leak then quantified as the fraction of caffeine-induced release? Please clarify and include the description in the Methods section. Why was 10mM caffeine used? Generally 3-5 mM caffeine produce the maximum calcium release from SR in cardiomyocytes. Use of the higher concentrations may activate calcium entry on hemi-channels, reported by some investigators.

We apologise for any confusion. SR Ca^{2+} leak was measured by quantifying Ca^{2+} sparks via confocal line scan. SR Ca^{2+} leak was calculated from Ca^{2+} spark frequency and Ca^{2+} spark size (Figure 4B). We have clarified this in the Methods section:

Page 7, line 122: "Images were acquired using Zen 2009 acquisition software and analysed using the ImageJ SparkMaster plug-in (Picht *et al.*, 2007). SR Ca^{2+} leak was calculated from Ca^{2+} spark frequency and Ca^{2+} spark size, as previously described (Fischer *et al.*, 2015)."

We agree with the Reviewer that caffeine can affect hemi-channels and indeed we have observed this behaviour in mouse cardiomyocytes (Fakuade *et al.*, 2021*b*). Interestingly, and in contrast, we found that this activity is rare in human atrial cardiomyocytes (Fakuade *et al.*, 2021*b*)and therefore we conclude that this does not play a significant role in the present study.

The protocol for the quantification of SR Ca^{2+} content by application of 10 mM caffeine is an established method published by Varro *et al.* (Varro *et al.*, 1993) and has been extensively and successfully used by us and others (Maier *et al.*, 2003; Stokke *et al.*, 2011; Voigt *et al.*, 2012; Fakuade *et al.*, 2021*a*). Therefore, we feel confident that our use of the method has provided accurate results.

3. Fig.1 shows ICaL current around 20 pA/pF, while Figs.5-6 show only ICaL amplitudes of 2-5 pA/pF. How can the authors explain these large difference in their results? Are the cells with larger calcium current autophosphrylated? The larger ICa density cells are more consistent with those reported for hiPSC-CMs in the literature! Did the measured Ca-transients reflect this large difference in ICa?

We appreciate the Reviewer's attention regarding the differences in $I_{Ca,L}$ current densities displayed in Figure 1 and Figures 5-6. We suggest that this discrepancy arises due to utilisation of two different techniques, rather than biological variability. It is important to note that in manual patch-clamp experiments, cardiomyocytes adhere to coated coverslips, enabling stable "attached" configuration (see Methods section), preserving membrane integrity and yielding higher current densities. In contrast, automated patch-clamp requires cellular suspension, leading to different "spatial confirmation" of cardiomyocytes. While absolute current density values differ, we observe that relative changes in response to blebbistatin are consistent between manual and automated patch-clamp experiments. We have noted this in the Potential Limitations section of the revised manuscript:

Page 14, line: 341: "We acknowledge there are differences in the absolute values of $I_{Ca,L}$ densities measured in manual and automated patch-clamp experiments. We attribute this to variation in the spatial configuration of the cell in the given technique, rather than intrinsic physiological variability. In manual patch-clamp, cells adhere to glass coverslips whereas

automated patch-clamp requires cellular suspension. Importantly, pharmacological response in both of the techniques was similar, supporting the validity of the findings."

4. Figure 2 shows greater dispersion of INCX in blebistatine treated cells consistent with larger Ca-transients. Why would increasing the calcium buffering result in larger Ca-transients? Does the dispersion arise from variance in Ica in different cells?

It appears there may be a misunderstanding about the impact of blebbistatin on calcium buffering. Blebbistatin, as demonstrated in our study, does not increase calcium buffering capacity but rather decreases the affinity of intracellular Ca^{2+} buffers (increased K_d). This reduction in buffer affinity leads to higher free Ca^{2+} levels during both diastole and systole, as observed in blebbistatin-treated cells (Figure 1B).

Regarding the greater dispersion of I_{NCX} observed in blebbistatin-treated cells, this could reflect the heterogeneity in the response of individual cardiomyocytes to changes in intracellular Ca^{2+} buffering upon blebbistatin treatment. Furthermore, our findings of increased SR Ca^{2+} leak and Ca^{2+} spark frequency with blebbistatin (Figure 4) indicate a more dynamic and variable intracellular Ca^{2+} environment.

5. Small-conductance Ca2+ activated potassium channels were shown to be present in both ventricles and atria, however functional activation of SK channels is significantly more prominent in atria. Increasing number of studies show the inhibition of SK channels as a potential antiarrhythmic treatment option in AF and other atrial arrhythmias. Did the Authors examine the effect of reduced Ca2+ buffering on the Ca2+ dependent K+ current in their atrial specific CMs. I would recommend doing such experiments.

We thank the Reviewer for this comment. SK channels have recently gained much attention in the literature. We would like to highlight the finding that trafficking of SK channels to the membrane is upregulated during atrial fibrillation (Heijman *et al.*, 2023). In contrast, the cardiomyocytes used in our study were healthy and were not subjected to tachypacing. Therefore we do not believe that SK channels play a large role in the findings of our current investigation and a detailed investigation into this would be beyond the scope of the study. However, we have added text to the Potential Limitations section, in order to address this point:

Page 14, line 347: "A further potential limitation in the current study concerns the contribution of SK channels, which have gained much attention in the literature recently. These channels, however, are thought to be more important in the setting of AF (Heijman *et al.*, 2023), whereas our study utilises healthy iPSC-CM."

5. The Authors have used blebbistatin in the concentration of 10 µM. Did the Authors test its dose-dependent effects on the intracellular Ca2+ signaling properties of hiPSC derived atrial CMs?

The main aim of this study was to draw awareness of potentially "unwanted" effects of blebbistatin when used as an experimental tool, for example in optical mapping studies to block contraction, thereby allowing efficient imaging. Blebbistatin is usually used in such studies at a concentration of 5-10 µM and there is evidence that 10 µM is optimal (Swift *et al.*,

2021) so we therefore chose this concentration for the present study. We feel that a full investigation of dose-dependent effects of blebbistatin is beyond the scope of our study.

6. iPSC derived cardiomyocytes have the advantage that they show spontaneous automaticity. Were these cells able to maintain spontaneous beating? If so, did the faster calcium dependent inactivation and the reduced IK1 result in faster beating rates?

This is a particularly interesting question in the field of iPSC-CMs. Indeed, spontaneous automaticity is a hallmark of iPSC-CMs, particularly, when cultured in a monolayer (Kim *et al.*, 2015; Casini *et al.*, 2017). In our study, iPSC-CMs initially exhibited spontaneous beating activity. However, over time, spontaneous beating frequency declined, consistent with previously reported observations linked to time-dependent maturation properties of iPSC-CMs (Lundy *et al.*, 2013; Seibertz *et al.*, 2023*b*). This unique property is primarily driven by increased function of I_{K1} and "improved/mature" electrophysiological profile (Kim *et al.*, 2015; Cyganek *et al.*, 2018; Seibertz *et al.*, 2023*b*). Interestingly, our experiments demonstrated reduced I_{K1} upon blebbistatin incubation, pointing to the fact that the observed change in the current density is indeed the effect of the drug.

Furthermore, it is important to note that the patch-clamp technique at the time of experiment may suppress spontaneous activity. Once seal is formed, cells are kept at a holding potential (- 80 mV) prior to the test pulse. This property can effectively interrupt any spontaneous depolarisations the cells may exhibit under baseline conditions.

7. Authors claim that the intracellular Ca2+ changes due to blebbistatin results in pro-arrhythmic changes. Did the Authors measure any indicators of arrhythmia for instance, EADs or DADs in these cells?

The readout for potential pro-arrhythmic activity in our study was SR Ca^{2+} leak in the form of Ca^{2+} sparks, as measured by confocal line scan. The main aim of the study as a whole was to raise awareness about effects of blebbistatin application such as during use as an experimental tool. Blebbistatin has no clinical use as a drug and so further and substantial experiments into EADs and DADs were not performed. Nevertheless, our recent publication demonstrated that using blebbistatin to reduce Ca^{2+} buffering increases the inducibility of arrhythmic behavior in an *ex vivo* mouse heart model (Fakuade *et al.*, 2024).

8. Did the authors check on calcium channel current kinetics and amplitude when Ba2+ was the charge carrier through the channel. This may provide some insight on the buffering issue, because acute application of Ba2+ containg solution lasting only a few seconds that fails to activate CICR and rise of cytosolic calcium transients may shed some light on the authors' proposal of compartmented calcium pools.

This is a very good idea, however, using Ba^{2+} to abolish SR Ca^{2+} release is not possible with syncropatch becauses Ca^{2+} is required for successful seal formation (Milligan *et al.*, 2009). Therefore, we used EGTA, a slow Ca^{2+} buffer, to prevent cytosolic Ca^{2+} transients and we believe this experiment reflects the Reviewer's intention. We found that EGTA does not abolish CDI completely. This might be due to the fact that Ca^{2+} influx through $I_{Ca,L}$ remains largely unchanged (due to the slow buffering kinetics of EGTA) and is therefore able to bring about CDI of $I_{Ca,L}$. Since under these conditions blebbistatin had no effect on CDI, we conclude

that blebbistatin does not effect the CDI caused by the Ca^{2+} entering via $I_{Ca,L}$. We have now added text to the Discussion to convey this:

Page 13, line 300: "The fact that EGTA, a slow buffer, did not completely abolish CDI in the presence or absence of blebbistatin, suggests that Ca^{2+} entering via $I_{Ca,L}$ can exert CDI and this is not affected by blebbistain, pointing towards compartmentalisation of Ca^{2+} signalling"

Minor: Starting almost every sentence with mean {plus minus} SEM in the figure legends is somewhat odd. I would recommend saying for example Quantification of CaT amplitude instead of Mean {plus minus} SEM CaT amplitude etc.

The figure legends have been revised accordingly.

References

Baudenbacher F, Schober T, Pinto JR, Sidorov VY, Hilliard F, Solaro RJ, Potter JD & Knollmann BC (2008). Myofilament Ca^{2+} sensitization causes susceptibility to cardiac arrhythmia in mice. *Journal of Clinical Investigation*; DOI: 10.1172/JCI36642.

Casini S, Verkerk AO & Remme CA (2017). Human iPSC-Derived Cardiomyocytes for Investigation of Disease Mechanisms and Therapeutic Strategies in Inherited Arrhythmia Syndromes: Strengths and Limitations. *Cardiovasc Drugs Ther* **31,** 325–344.

Cyganek L, Tiburcy M, Sekeres K, Gerstenberg K, Bohnenberger H, Lenz C, Henze S, Stauske M, Salinas G, Zimmermann W-H, Hasenfuss G & Guan K (2018). Deep phenotyping of human induced pluripotent stem cell–derived atrial and ventricular cardiomyocytes. *JCI Insight*; DOI: 10.1172/jci.insight.99941.

Dou Y, Arlock P & Arner A (2007). Blebbistatin specifically inhibits actin-myosin interaction in mouse cardiac muscle. *Am J Physiol Cell Physiol* **293,** C1148-53.

Fakuade FE et al. (2021*a*). Altered atrial cytosolic calcium handling contributes to the development of postoperative atrial fibrillation. *Cardiovasc Res* **117,** 1790–1801.

Fakuade FE et al. (2024). Impaired Intracellular Calcium Buffering Contributes to the Arrhythmogenic Substrate in Atrial Myocytes From Patients With Atrial Fibrillation. *Circulation* **150,** 544–559.

Fakuade FE, Tomsits P & Voigt N (2021*b*). Connexin hemichannels in atrial fibrillation: orphaned and irrelevant? *Cardiovasc Res* **117,** 4–6.

Findlay I (2004). Physiological modulation of inactivation in L-type Ca^{2+} channels: one switch. *J Physiol* **554,** 275–283.

Fischer TH, Herting J, Mason FE, Hartmann N, Watanabe S, Nikolaev VO, Sprenger JU, Fan P, Yao L, Popov A-F, Danner BC, Schöndube F, Belardinelli L, Hasenfuss G, Maier LS & Sossalla S (2015). Late I_{Na} increases diastolic SR-Ca^{2+}-leak in atrial myocardium by activating PKA and CaMKII. *Cardiovasc Res* **107,** 184–196.

Grandi E, Morotti S, Ginsburg KS, Severi S & Bers DM (2010). Interplay of voltage and Ca-dependent inactivation of L-type Ca current. *Prog Biophys Mol Biol* **103,** 44–50.

Heijman J, Zhou X, Morotti S, Molina CE, Abu-Taha IH, Tekook M, Jespersen T, Zhang Y, Dobrev S, Milting H, Gummert J, Karck M, Kamler M, El-Armouche A, Saljic A, Grandi E, Nattel S & Dobrev D (2023). Enhanced Ca^{2+}-Dependent SK-Channel Gating and Membrane Trafficking in Human Atrial Fibrillation. *Circ Res* **132,** e116–e133.

Kim JJ, Yang L, Lin B, Zhu X, Sun B, Kaplan AD, Bett GCL, Rasmusson RL, London B & Salama G (2015). Mechanism of automaticity in cardiomyocytes derived from human induced pluripotent stem cells. *J Mol Cell Cardiol* **81,** 81–93.

Knollmann B & Bers D (2024). Buffering the Heart Against Atrial Fibrillation. *Circulation*.

Lundy SD, Zhu W-Z, Regnier M & Laflamme MA (2013). Structural and functional maturation of cardiomyocytes derived from human pluripotent stem cells. *Stem Cells Dev* **22,** 1991–2002.

Mahajan A, Shiferaw Y, Sato D, Baher A, Olcese R, Xie L-H, Yang M-J, Chen P-S, Restrepo JG, Karma A, Garfinkel A, Qu Z & Weiss JN (2008). A rabbit ventricular action potential model replicating cardiac dynamics at rapid heart rates. *Biophys J* **94,** 392–410.

Maier LS, Zhang T, Chen L, DeSantiago J, Brown JH & Bers DM (2003). Transgenic CaMKIIdeltaC overexpression uniquely alters cardiac myocyte Ca^{2+} handling: reduced SR Ca^{2+} load and activated SR Ca2+ release. *Circ Res* **92,** 904–911.

Milligan CJ et al. (2009). Robotic multiwell planar patch-clamp for native and primary mammalian cells. *Nat Protoc* **4,** 244–255.

Picht E, Zima A V, Blatter LA & Bers DM (2007). SparkMaster: automated calcium spark analysis with ImageJ. *Am J Physiol Cell Physiol* **293,** C1073-81.

Schober T, Huke S, Venkataraman R, Gryshchenko O, Kryshtal D, Hwang HS, Baudenbacher FJ & Knollmann BC (2012). Myofilament Ca Sensitization Increases Cytosolic Ca Binding Affinity, Alters Intracellular Ca Homeostasis, and Causes Pause-Dependent Ca-Triggered Arrhythmia. *Circ Res* **111,** 170–179.

Seibertz F et al. (2023*a*). Atrial fibrillation-associated electrical remodelling in human induced pluripotent stem cell-derived atrial cardiomyocytes: a novel pathway for antiarrhythmic therapy development. *Cardiovasc Res* **119,** 2623–2637.

Seibertz F et al. (2023*b*). Electrophysiological and calcium-handling development during long-term culture of human-induced pluripotent stem cell-derived cardiomyocytes. *Basic Res Cardiol* **118,** 14.

Seibertz F, Rapedius M, Fakuade FE, Tomsits P, Liutkute A, Cyganek L, Becker N, Majumder R, Clauß S, Fertig N & Voigt N (2022). A modern automated patch-clamp approach for high throughput electrophysiology recordings in native cardiomyocytes. *Commun Biol* **5,** 969.

Smith GL & Eisner DA (2019). Calcium Buffering in the Heart in Health and Disease. *Circulation* **139,** 2358–2371.

Stokke MK, Briston SJ, Jølle GF, Manzoor I, Louch WE, Øyehaug L, Christensen G, Eisner DA, Trafford AW, Sejersted OM & Sjaastad I (2011). Ca^{2+} wave probability is determined by the balance between SERCA2-dependent Ca^{2+} reuptake and threshold SR Ca^{2+} content. *Cardiovasc Res* **90,** 503–512.

Swift LM, Kay MW, Ripplinger CM & Posnack NG (2021). Stop the beat to see the rhythm: excitation-contraction uncoupling in cardiac research. *Am J Physiol Heart Circ Physiol* **321,** H1005–H1013.

Varro A, Negretti N, Hester SB & Eisner DA (1993). An estimate of the calcium content of the sarcoplasmic reticulum in rat ventricular myocytes. *Pflugers Arch* **423,** 158–160.

Voigt N, Li N, Wang Q, Wang W, Trafford AW, Abu-Taha I, Sun Q, Wieland T, Ravens U, Nattel S, Wehrens XHT & Dobrev D (2012). Enhanced Sarcoplasmic Reticulum Ca^{2+} Leak and Increased Na^{+}-Ca^{2+} Exchanger Function Underlie Delayed Afterdepolarizations in Patients With Chronic Atrial Fibrillation. *Circulation* **125,** 2059–2070.

Voigt N, Trausch A, Knaut M, Matschke K, Varró A, Van Wagoner DR, Nattel S, Ravens U & Dobrev D (2010). Left-to-Right Atrial Inward Rectifier Potassium Current Gradients in Patients With Paroxysmal Versus Chronic Atrial Fibrillation. *Circ Arrhythm Electrophysiol* **3,** 472–480.

Dear Professor Voigt,

Re: JP-RP-2025-287545R1 "Blebbistatin reduces calcium buffering in cardiomyoctes: Consequences for cellular electrophysiology" by Izzatullo Sobitov, Katharina Ritzenhoff, Marie Gaulrapp, Lea Becker, Aiste Liutkute, Fitzwilliam Seibertz, Funsho E. Fakuade, Fleur E. Mason, and Niels Voigt

We are pleased to tell you that your paper has been accepted for publication in The Journal of Physiology.

The last Word (or similar) version of the manuscript provided will be used by the Production Editor to prepare your proof. When this is ready you will receive an email containing a link to Wiley's Online Proofing System. The proof should be thoroughly checked and corrected as promptly as possible.

Authors should note that it is too late at this point to offer corrections prior to proofreading. Major corrections at proof stage, such as changes to figures, will be referred to the Editors for approval before they can be incorporated. Only minor changes, such as to style and consistency, should be made at proof stage. Changes that need to be made after proof stage will usually require a formal correction notice.

All queries at proof stage should be sent to: TJP@wiley.com.

Following copyediting and typesetting the accepted version will be published online.

Yours sincerely,

Bjorn Knollmann
Senior Editor
The Journal of Physiology

IMPORTANT POINTS TO NOTE FOLLOWING YOUR PAPER'S ACCEPTANCE:

If you would like to receive our 'Research Roundup', a monthly newsletter highlighting the cutting-edge research published in The Physiological Society's family of journals (The Journal of Physiology, Experimental Physiology, Physiological Reports, The Journal of Nutritional Physiology and The Journal of Precision Medicine: Health and Disease), please click this link, fill in your name and email address and select 'Research Roundup':
https://www.physoc.org/journals-and-media/membernews

• TRANSPARENT PEER REVIEW POLICY: To improve the transparency of its peer review process, The Journal of Physiology publishes online as supporting information the peer review history of all articles accepted for publication. Readers will have access to decision letters, including Editors' comments and referee reports, for each version of the manuscript as well as any author responses to peer review comments. Referees can decide whether or not they wish to be named on the peer review history document.

• You can help your research get the attention it deserves! Check out Wiley's free Promotion Guide for best-practice recommendations for promoting your work at: www.wileyauthors.com/eeo/guide. You can learn more about Wiley Editing Services which offers professional video, design, and writing services to create shareable video abstracts, infographics, conference posters, lay summaries, and research news stories for your research at: www.wileyauthors.com/eeo/promotion.

• IMPORTANT NOTICE ABOUT OPEN ACCESS: To assist authors whose funding agencies mandate public access to published research findings sooner than 12 months after publication, The Journal of Physiology allows authors to pay an Open Access (OA) fee to have their papers made freely available immediately on publication.

The Corresponding Author will receive an email from Wiley with details on how to register or log-in to Wiley Authors Services where you will be able to place an order.

• You can check if your funder or institution has a Wiley Open Access Account here: https://authorservices.wiley.com/author-resources/Journal-Authors/licensing-and-open-access/open-access/author-compliance-tool.html.

EDITOR COMMENTS

Reviewing Editor:

The referees agree that the revised manuscript is expected to have a significant impact on the field.

Senior Editor:

The manuscript is now acceptable. Thank you for your excellent contribution!

REFEREE COMMENTS

Referee #1:

The authors present a responsive revised manuscript. The concerns in my original review have been directly and thoughtfully addressed. This is a meaningful study convincingly demonstrating that blebbistatin alters Ca buffering in iPSC-aCMs.

Referee #2:

I have no other comments.
